# Convergent neuroimmune signaling underlying rapid antidepressant response to ketamine and psychedelics

**DOI:** 10.1038/s41380-026-03777-z

**Published:** 2026-07-28

**Authors:** Gregory H. Jones, Jessica R. Gilbert, Jenessa N. Johnston, Nirmala Akula, Anton Schulmann, Miranda Arakelian, Shiyong Peng, Peixiong Yuan, Ewurakua A. Winful, Mani Yavi, Brandi Quintanilla, Abdel Elkahloun, Ruin Moaddel, Ioline D. Henter, Dede Greenstein, Rodrigo Machado-Vieira, Christopher M. Bartley, Bashkim Kadriu, Moran Amit, Katy Rezvani, Mark D. Kvarta, Francis J. McMahon, Carlos A. Zarate

**Affiliations:** 1 Experimental Therapeutics & Pathophysiology Branch, Division of Intramural Research Program, National Institute of Mental Health, Bethesda, MD USA; 2 Department of Psychiatry, The University of Texas MD Anderson Cancer Center, Houston, TX USA; 3 Human Genetics Branch, Intramural Research Program, National Institute of Mental Health, Bethesda, MD USA; 4 Department of Psychiatry and Behavioral Sciences, University of Texas Health Science Center, Houston, TX USA; 5 Laboratory of Cancer Genetics, National Human Genome Research Institute, National Institutes of Health, Bethesda, MD USA; 6 Biomedical Research Center, National Institute on Aging, National Institutes of Health, Baltimore, MD USA; 7 Translational Immunopsychiatry Unit, National Institute of Mental Health, Bethesda, MD USA; 8 Department of Head and Neck Surgery, The University of Texas MD Anderson Cancer Center, Houston, TX USA; 9 Department of Stem Cell Transplantation and Cellular Therapy, The University of Texas MD Anderson Cancer Center, Houston, TX USA

**Keywords:** Stem cells, Neuroscience, Predictive markers, Depression

## Abstract

Despite distinct receptor targets, both ketamine and serotonergic psychedelics produce a rapid clinical response and share biological signatures that suggest convergence on common downstream molecular mediators. To identify shared biomarkers of rapid antidepressant response, this study integrated CSF proteomics from healthy volunteers (HVs) who received intravenous ketamine with transcriptomic analyses from induced pluripotent stem cells (iPSCs) derived from participants with treatment-resistant depression (TRD) and HVs; iPSCs were treated with ketamine, its metabolite (2 *R*,6 *R*)-hydroxynorketamine, lysergic acid diethylamide (LSD), or psilocybin. Multimodal clinical characterization (transcriptomics (*n* = 16 TRD; 11 HV), magnetoencephalography (MEG) (*n* = 30 TRD; 25 HV), and plasma cytokines (*n* = 39 TRD; 25 HV) were also performed on TRD and HV participants who received a single dose of intravenous ketamine (0.5 mg/kg) or placebo. Conserved immune pathways were identified across CSF and iPSC neurons with interleukin-15 (IL)-15 and monocyte chemoattractant protein-1 (MCP-1) emerging as key regulatory hubs. Transcriptomically, in whole blood, ketamine responders exhibited decreased IL-15 and elevated B-cell signaling pathways at baseline that were reversed post-treatment. At the protein level, plasma IL-7 levels (primary B-cell driver) correlated with baseline MEG gamma power, reaching brain-wide significance across all participants (main effect p_FDR_ < 0.05). The association was most pronounced in the TRD participants across subcortical regions (diagnosis x IL-7 p_FDR_ < 10^-14^). Post-ketamine, the TRD IL-7–gamma relationship inverted, paralleling widespread gamma power reductions throughout default-mode network regions (session x IL-7 p_clc_ < 0.05). In mixed-effects models, cytokine ratios linked to IL-7/IL-15 signaling predicted antidepressant response (IL-4/interferon gamma (IFN-γ) p_FDR_ < 0.041) and non-response (MCP-1/IL-7 p_FDR_ < 0.009), suggesting that rebalancing within the IL-7/IL-15 axis may contribute to therapeutic efficacy. Clinicaltrials.gov identifier: NCT00088699; NCT02484456.

## Introduction

Rapid-acting antidepressants (RAADs) such as ketamine and psilocybin have transformed the treatment landscape for treatment-resistant depression (TRD), producing robust clinical response in ~40-50% of individuals with TRD within a day after the first administration [1,2]. However, ketamine’s effects are short-lived, even after repeated administrations, with a median time to relapse after stopping ketamine of 18 days [3]. Psilocybin assisted therapy, while effective, requires substantial mental health resources [4] and is difficult to test in blinded settings [5,6], among other issues [7]. These translational challenges underscore the critical need to identify the biological mechanisms underlying rapid antidepressant effects, which could inform the development of more durable and accessible treatments.

Despite distinct primary receptor targets, both ketamine and psilocybin elicit similar biological correlates of treatment response, including increased prefrontal cortical glutamate levels [8–11], increased magnetoencephalographic (MEG) signal entropy that correlates with the intensity of subjective experience [12], and mechanistic target of rapamycin complex 1 (mTORC1)-sensitive changes in structural and functional plasticity mediated via brain-derived neurotrophic factor (BDNF) signaling [13–15]. These observations suggest that, despite highly distinct subjective effects, upstream pathways, and pharmacodynamic properties, ketamine, psilocybin, and other putative RAADs may engage convergent downstream molecular mediators that remain incompletely characterized [16].

Emerging evidence points to neuroimmune modulation as a potential convergent mechanism. Depression has long been associated with chronic low-grade inflammation, both peripherally (e.g., elevated levels of C-reactive protein (CRP), interleukin-6 (IL-6), tumor necrosis factor alpha (TNF-α), and other proinflammatory cytokines [17]), and centrally (e.g., elevated cerebrospinal fluid (CSF) levels of IL-6 and TNF-α as well as translocator protein identified via PET) [18]. Heightened inflammatory tone (CRP levels [19]) has also been a well-established predictor of TRD. Furthermore, the mTORC1 pathway—central to both ketamine and psilocybin’s synaptic effects—serves as a master regulator of immune cell metabolism and function [20,21], suggesting that RAADs may jointly regulate neuroimmune function. Consistent with this, direct mTORC1 inhibition via intracerebroventricular administration of rapamycin reliably blocked RAAD-induced neuroplasticity and ketamine’s antidepressant behavioral effects in preclinical models [14, 22–24]. Peripheral rapamycin administration, however, has produced mixed results [25,26], and one randomized crossover trial found that pretreatment with oral rapamycin (6 mg) paradoxically tripled ketamine response rates [27]. Whether oral rapamycin achieves meaningful CNS mTORC1 inhibition at this dose is uncertain [28,29]. Neuroimaging or longitudinal biomarkers apart from drug levels have not been reported [27]. The only cited human evidence for CNS engagement at this dose derives from rapamycin used daily as salvage therapy in glioblastoma, a population and tumor type intentionally selected to amplify potential CNS mTOR signals [30], limiting generalizability to healthy parenchyma. Collectively, these considerations underscore the need to build on critical findings by integrating peripheral immune and central surrogate measures in humans to advance our mechanistic understanding of RAADs.

**Fig. 1:**
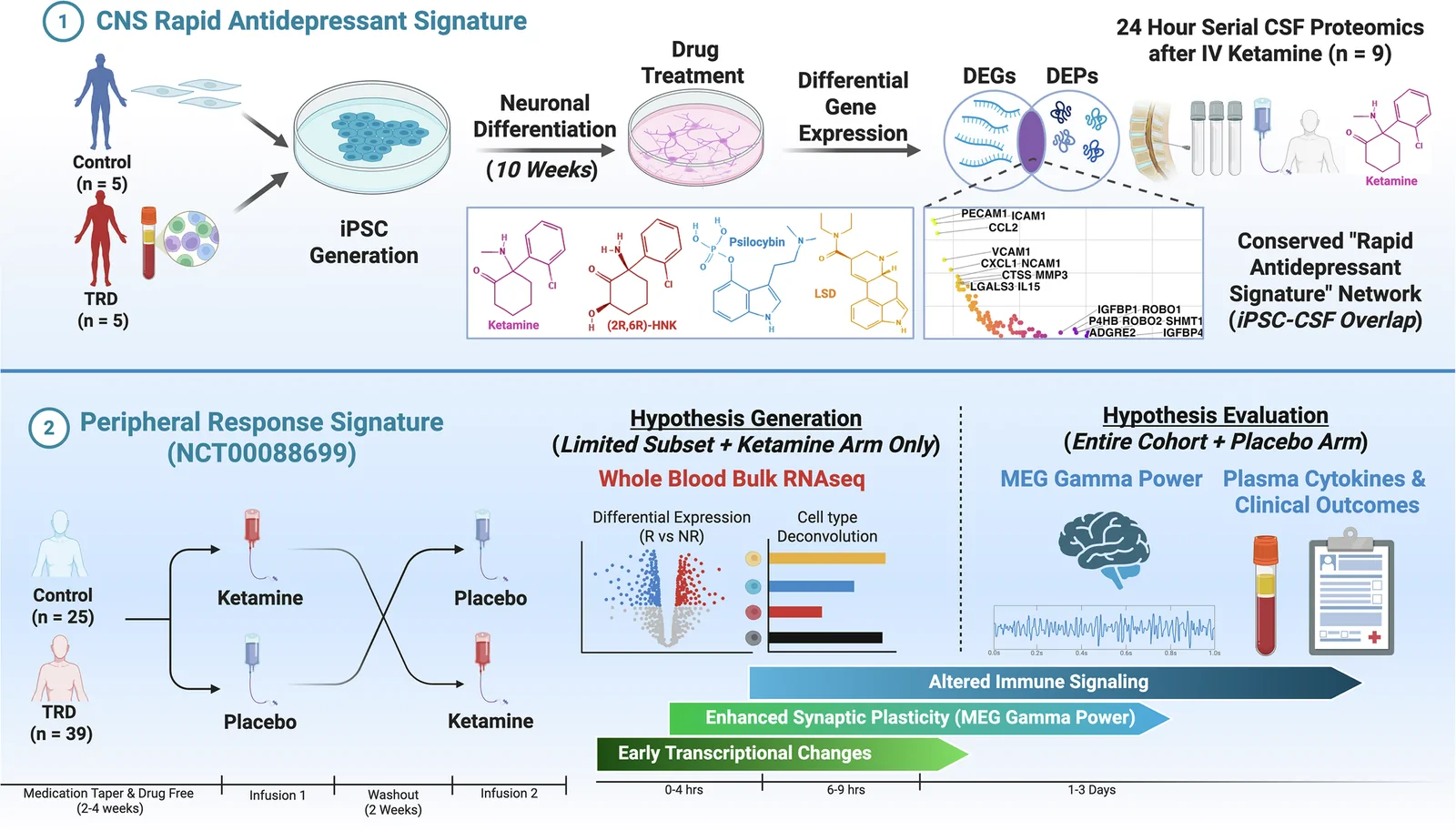
Experimental–clinical workflow for rapid antidepressant signatures. **(1) Central nervous system (CNS) rapid antidepressant signatures**. Patient- and control-derived induced pluripotent stem cells (iPSCs) were differentiated into cortical neurons, exposed to ketamine, (2*R*,6 *R*)-hydroxynorketamine (HNK), lysergic acid diethylamide (LSD), or psilocybin, and profiled by bulk RNA-seq. Differentially expressed genes (DEGs) were compared with serial CSF proteomics after IV ketamine to define a conserved molecular signature and network. **(2) Peripheral response signatures**. In a randomized crossover trial (NCT00088699), participants with treatment-resistant depression (TRD; *n* = 39) and healthy volunteers (HVs; *n* = 25) received ketamine or a placebo infusion. A subset of participants (*n* = 16 TRD, *n* = 11 HV) underwent whole-blood RNA-seq to identify DEGs among groups and treatments, and the full cohort was assessed with magnetoencephalography (MEG) gamma power, plasma biomarkers, and clinical outcomes. Mixed-effects models tested the associations between treatment, biomarkers, and change in symptoms.

This study sought to address that gap by examining molecular mechanisms underlying two established RAADs—ketamine and psilocybin—and two emerging RAADs: (2 *R*,6 *R*)-hydroxynorketamine ((2 *R*,6 *R*)-HNK), a longer-lasting, non-dissociative ketamine metabolite currently in Phase II trials (NCT06511908), and lysergic acid diethylamide (LSD, MM120), an ergoline derivative serotonergic psychedelic now in Phase III trials for major depressive disorder (MDD) (NCT06941844). Transcriptomic data were integrated from induced pluripotent stem cell (iPSC)-derived neurons treated with these four compounds alongside serial CSF proteomics from participants treated with intravenous ketamine (Fig. 1**, top**) [31]. This approach enabled identification of conserved molecular signatures that could be corroborated through multimodal clinical characterization (Fig. 1**, bottom**). Specifically, the study incorporated whole blood transcriptomics, plasma cytokine profiling, and MEG gamma power (a well-established, cross-species biomarker of RAAD response) [32] collected from a previous double-blind, placebo-controlled, randomized crossover study (NCT00088699) of participants with TRD (*n* = 39) and healthy volunteers (HVs) (*n* = 25) who received a single intravenous infusion of ketamine (0.5 mg/kg) or saline placebo. Collectively, this work sought to identify biomarkers conserved across pharmacologically distinct RAADs, offering mechanistic insights into rapid antidepressant action and potential predictors of treatment response.

## Materials and methods

### Comparison of iPSC-derived cortical neurons and CSF proteomics

Peripheral blood mononuclear cells (PBMCs) from participants with TRD (*n* = 5) and fibroblasts from HVs (*n* = 5) (all female samples) were obtained for iPSC reprogramming after obtaining written informed consent (NCT02484456); these were differentiated to cortical neurons using the STEMdiff™ protocol (StemCell Technologies) followed by 10-week maturation. Neurons were exposed for 24 h to vehicle, (2 *R*,6 *R*)-HNK (1 μM), ketamine (1 μM), LSD (10 μM), or psilocybin (10 μM) based on literature-supported ranges [14, 33]. Bulk RNA sequencing was performed and aligned to GRCh38 (v113) using STAR-featureCounts [34]. Differential expression was conducted using Dream/Limma [35] after filtering low-abundance genes (CPM < 0.1 in ≥5 samples); *variancePartition* guided covariate selection [35,36], with treatment condition as a fixed effect and diagnosis, sequencing batch, and individual donor as random effects. iPSC differentially expressed genes (DEGs) were compared with differentially expressed proteins (DEPs) from serial CSF proteomics previously collected from nine HVs who received IV ketamine (Olink Explore platform; six samples per volunteer over 24 h) [31]. Network analysis of overlapping iPSC-DEGs/CSF-DEPs used STRING [37] and Cytoscape [38] for topology and hub identification. The full protocol and methods are provided in the Supplement.

### Clinical ketamine crossover trial

A post-hoc analysis was performed on data (whole blood RNAseq, plasma cytokines, clinical outcomes, and MEG) drawn from a previous randomized, placebo-controlled crossover study of TRD (*n* = 39) and HV (*n* = 25) participants who received a single IV ketamine (0.5 mg/kg) versus saline infusion (NCT00088699). TRD participants met DSM-IV criteria for major depression, had Montgomery-Asberg Depression Rating Scale (MADRS) scores ≥20, and had failed to respond to at least two adequate antidepressant trials (see Supplement for additional study details). Whole-blood RNA was collected (Paxgene) at baseline and four hours post-ketamine from 27 participants (*n* = 16 TRD; *n* = 11 HV), with globin-ribosomal-depletion (Illumina Ribo-Zero Plus) prior to sequencing. Alignment (HISAT2 [39]; Ensembl version 113) and differential expression (Dream/Limma [35]) were implemented, using mixed models with group x time interaction, RNA integrity number (RIN), sex, and race (self-identified) as fixed effects (based on *variancePartition*) [35,36] and participant as a random effect. Groups included HVs, ketamine non-responders, and ketamine responders (defined as ≥50% reduction in MADRS score at 24 h). Ingenuity pathway analysis (IPA) [40] was conducted on ketamine responder versus non-responder contrasts. *xCell* [41] estimated cell-type fractions, which were compared with absolute and relative lymphocyte counts from available complete blood cell counts (CBCs) collected during pre-trial screening (*n* = 20/27) using established methods [42]. *BayesPrism* [43] modeled cell-type-specific differential expression using a single-cell PBMC reference [44] with the same differential expression model as above.

For cytokines, plasma was collected at baseline, four hours, one day, and three days post-ketamine/placebo and analyzed via Bio-Plex 27-plex assay (BioRad). Analytes with >25% samples below detection (*n* = 7/27) were excluded. Linear mixed effects models included cytokine×treatment interaction, time, infusion order, age, body-mass index (BMI), sex, and baseline MADRS as fixed effects. Because crossover designs randomize patients to sequences rather than treatments, subject-average baseline scores were included as an additional covariate to address cross-level bias, as recommended by Kenward and Roger for these study designs [45]. Standardized effect sizes for each cytokine interaction were calculated using the *Effectsize* package in *R* [46]. A sensitivity analysis was also conducted to assess the influence of comorbid inflammatory illnesses and as-needed anti-inflammatory medications taken by patients during the study. Chronic use was an exclusionary criterion for enrollment. See Supplement for complete methodology and results.

Resting-state MEG data were acquired at baseline and six to nine hours post-infusion (*n* = 30 TRD, *n* = 25 HV), using a CTF 275-channel system. Source-localized gamma power (30-50 Hz) was reconstructed via SAM beamforming [47]. Linear mixed-effects models were analyzed in Analysis of Functional NeuroImages (AFNI) [48] to test the effects of IL-7 or monocyte chemoattractant protein-1 (MCP-1), accounting for diagnosis and session (baseline, post-ketamine, post-placebo). See Supplement for full acquisition parameters.

### Statistical analysis

All statistical tests were two-sided unless otherwise specified. For linear mixed-effects models of cytokine x clinical outcome associations (lme4/lmerTest) (version 4.5.0), Kenward-Roger approximations were used for degrees of freedom and *p*-values. Model assumptions, including normality of residuals, homoscedasticity, absence of influential outliers, and absence of multicollinearity were evaluated using the performance package in R [49] and in AFNI’s 3dLMEr residual diagnostics [48]; diagnostics supported the adequacy of model fits (see Supplementary Data File). Variance was inspected, and variance stabilizing transformations were applied as appropriate. Cytokine values were log-transformed prior to modeling to reduce positive skewness and z-scored to enable cross-cytokine comparison with MADRS scores as the continuous outcome. Center values are reported as means with standard deviations for continuous demographic and clinical variables (Tables.S1,S3,S4). For transcriptomic analyses (Dream/Limma), empirical Bayes moderated *t*-statistics were used, which inherently account for variance stabilization across genes; low-abundance genes were filtered (CPM > 0.1 in ≥5 samples) prior to testing. Multiple comparison corrections were applied as follows: false discovery rate (FDR; Benjamini-Hochberg) for CSF proteomics, cell-type deconvolution (xCell), cytokine ratio-MADRS interactions, and MEG source maps (cluster-level correction via AFNI). Individual gene-level transcriptomic contrasts used uncorrected p < 0.05 with fold-change >1.2 for hypothesis generation, with corroboration required across independent datasets as detailed in the Discussion. No formal power analysis was performed, as this was a post-hoc analysis of completed trials (NCT00088699; NCT02484456) [50]; sample sizes reflect the maximum available participants for each data modality.

## Results

### Overlap of iPSC-derived neuronal and CSF protein changes with RAADs

The first question of interest was whether transcriptional responses were shared across pharmacologically distinct RAADs and to what extent they overlapped with human CSF changes with the most clinically validated RAAD (ketamine). Within the iPSC-derived neurons, pairwise correlations (*z*-statistic) for DEGs with all four RAADs were high (*n* = 3157; r = 0.7-0.88; all p_fdr_ < 2e^-16^) (Fig. S1). Given this high correlation across RAADs, a conserved signature within the iPSCs was developed, and iPSC differential gene expression was descriptively compared with serial CSF proteomics collected from HVs after a single ketamine infusion (*n* = 9; six draws each over 24 h) [31]. This design—which used CSF as a direct readout of brain-adjacent, protein-level changes—was intended as a reciprocal control. In turn, overlap with iPSC neurons allowed the isolation of probable drug-induced immune changes from inflammatory responses known to be provoked by indwelling spinal catheters [51], thus maximizing the utility of both datasets. Given the differences between datasets, results are presented descriptively without statistical testing.

**Fig. 2:**
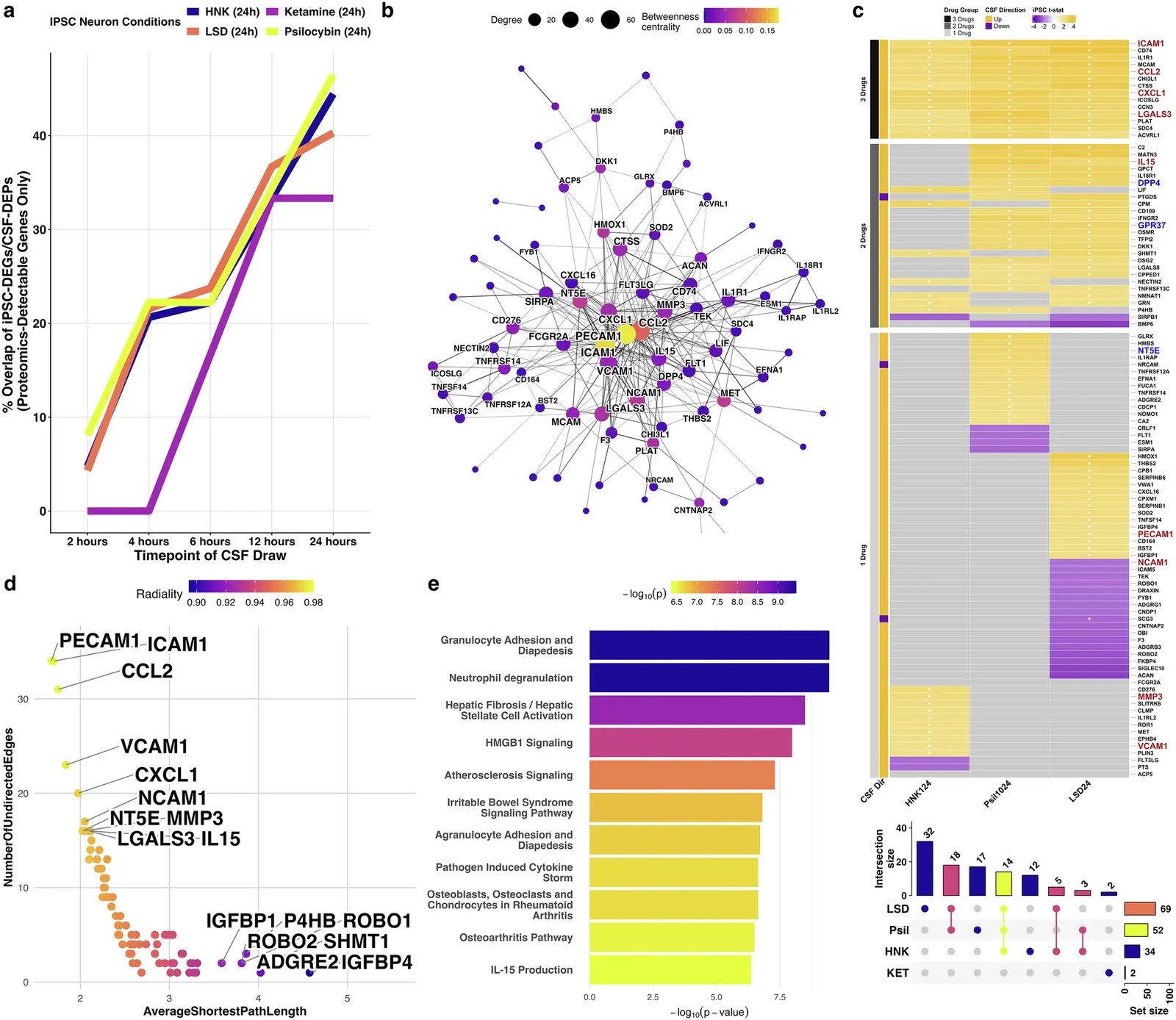
Temporal dynamics and cross-platform evaluation of rapid-acting antidepressants (RAADs) revealed shared molecular signatures. **a** Descriptive comparison of overlapping differentially expressed genes (DEGs) between ketamine-treated CSF proteomics and induced pluripotent stem cell (iPSC) neurons treated with ketamine, (2 *R*,6 *R*)-hydroxynorketamine (HNK), lysergic acid diethylamide (LSD), or psilocybin. Overlap percentages were calculated relative to the DEGs captured by the CSF proteomics platform as a common denominator across conditions. STRING network analysis identified 103 core proteins differentially expressed across the four rapid-acting antidepressants (RAADs) collectively in iPSC neurons that aligned with ketamine-induced CSF protein changes (complete gene list in Table.S2). **b** Rescaled STRINGdb network plot (default parameters) displaying major regulatory hubs within the 103-protein network (full plot shown in detail in Fig.S2). **c, top** Heatmap displaying overlap of CSF differentially expressed proteins (DEPs) with RAAD differential gene expression across conditions. Values represent iPSC neuron *t*-statistics for the 103-protein core signature, stratified by the number of RAAD conditions in which each overlapping iPSC-CSF marker was differentially expressed. Columns represent each iPSC drug condition at the 24-hour timepoint; grey cells indicate non-significant values for that drug (p ≥ 0.05). White dots indicate markers with 100% agreement in the direction of expression between iPSC conditions and CSF. Highlighted gene labels denote core adenosine signaling molecules (blue: NT5E, GPR37, DPP4) and top immune markers in the network analysis (red). **c, bottom** UpSet plot showing the degree of overlap in DEGs across the four RAAD conditions (LSD, psilocybin, (2 *R*,6 *R*)-HNK, ketamine). Bar height indicates the number of genes unique to each intersection, with dot connectivity patterns below denoting which drug conditions contribute to each set. Right bars indicate total set size per condition. **d** Network topology analysis of the 103-protein core signature using Cytoscape, displaying node connectivity (radiality), edge relationships, and shortest path lengths. Two distinct functional modules emerged: a highly connected primary module (upper left) and a peripheral secondary module (lower right). Labeled nodes represent proteins with the highest connectivity scores and/or shortest path lengths, indicating central placement within their respective hubs. **e** Ingenuity Pathway Analysis (IPA) functional enrichment of the core 103 protein signature.

When restricting analysis to iPSC DEGs with corresponding Olink-detectable proteins, substantial overlap was observed between ketamine-induced changes in the CSF and alterations in iPSCs after all four RAADs. Specifically, of the iPSC-DEGs with corresponding Olink proteins (i.e., both DEPs and non-DEPs), the following percentages were also identified as CSF DEPs: 46.5% (46/99) for psilocybin, 44.4% (28/63) for (2 *R*,6 *R*)-HNK, 40.3% (56/139) for LSD, and 33.3% (2/6) for ketamine (Fig. 2a).

Building on these results, 103 unique proteins were aggregated that were differentially expressed in CSF post-ketamine and also altered in iPSC neurons after treatment by at least one RAAD (Figs. 2b, 2c, S2). Eighty-four percent of these proteins came from matched 24-hour comparisons. Of the 103 proteins, 14 were shared by three drugs, 26 by two drugs, and the remainder were drug-specific. Importantly, very few proteins were exclusive to glutamatergic RAADs alone (ketamine and (2 *R*,6 *R*)-HNK); 86% (89/103) were also identified by at least one serotonergic psychedelic in iPSCs, including all proteins found by two or three drugs **(**Fig.2c, Table.S2).

To determine their functional relationship, network analysis was performed using STRING (for shared protein-protein interactions) and Cytoscape (for network connectivity) on all 103 markers (Fig. 2d). Stratification for radiality (the centrality of each marker), undirected edges (overall connectedness), and path length (steps between markers) revealed two distinct modules within this network. The primary module comprised a highly integrated network of immune signaling molecules with IL-15, MCP-1/CCL2, chemokine ligand 1 (CXCL1), and several cellular adhesion molecules (PECAM1, ICAM, VCAM, NCAM) serving as core regulators. A second, more peripheral module contained proteins related to insulin signaling (IGFBP1/IGFBP4/P4HB), synaptic plasticity (ROBO1/2), and folate metabolism (SHMT1) (Fig. 2d). This organization implies that immune signaling was centrally positioned within the broader metabolic and synaptic pathway networks associated with the effects of RAADs. IPA showed strong enrichment for immune and inflammatory pathways, together with multiple bone-metabolism-related programs and IL-15 production (Fig. 2e).

Collectively, these findings suggest substantial molecular convergence across pharmacologically distinct RAADs with immune signaling pathways. This cross-class overlap suggests that diverse RAADs may engage shared downstream pathways despite their distinct primary targets. The correspondence between iPSC neuronal responses and human CSF proteomics also supports the utility of this platform for screening novel RAAD candidates.

### Peripheral evaluation of IPSC-CSF immune signatures in association with clinical response

Given the strong enrichment of immune pathways in the overlapping iPSC-CSF network, the study next examined whether these central immune changes were also detectable in the periphery and whether they were linked to clinical response rather than general pharmacological exposure. Whole-blood RNA sequencing and plasma multiplex cytokine levels were examined for 39 TRD and 25 HV participants enrolled in a previously completed randomized, double-blind, placebo-controlled, crossover trial of ketamine versus saline placebo (see Methods); TRD participants were hospitalized and tapered off all antidepressants prior to starting the study. The cohort was severely treatment-refractory, having failed to respond to almost five adequate antidepressant trials on average, and demonstrated negligible placebo response (see Supplement for a full description of treatment-resistance history (Table.S3) and breakdown of cohorts by modality (blood-RNA, plasma cytokines, MEG (Table.S4)).

Whole-blood transcriptomics were performed on a subset of participants (*n* = 16 TRD, *n* = 11 HVs) during the ketamine arm of the crossover trial, with samples collected at baseline and at four hours post-infusion. This timepoint was selected to capture early transcriptional shifts that likely preceded other physiologic alterations differentiating responders ( ≥ 50% reduction in MADRS score) at the time of peak clinical antidepressant effects (24 h) [52]. This provided an opportunity to generate focused hypotheses that could then be substantiated at the plasma cytokine and neuroimaging levels (which incorporated both the placebo arm and timepoints out to three days post-infusion).

**Fig. 3:**
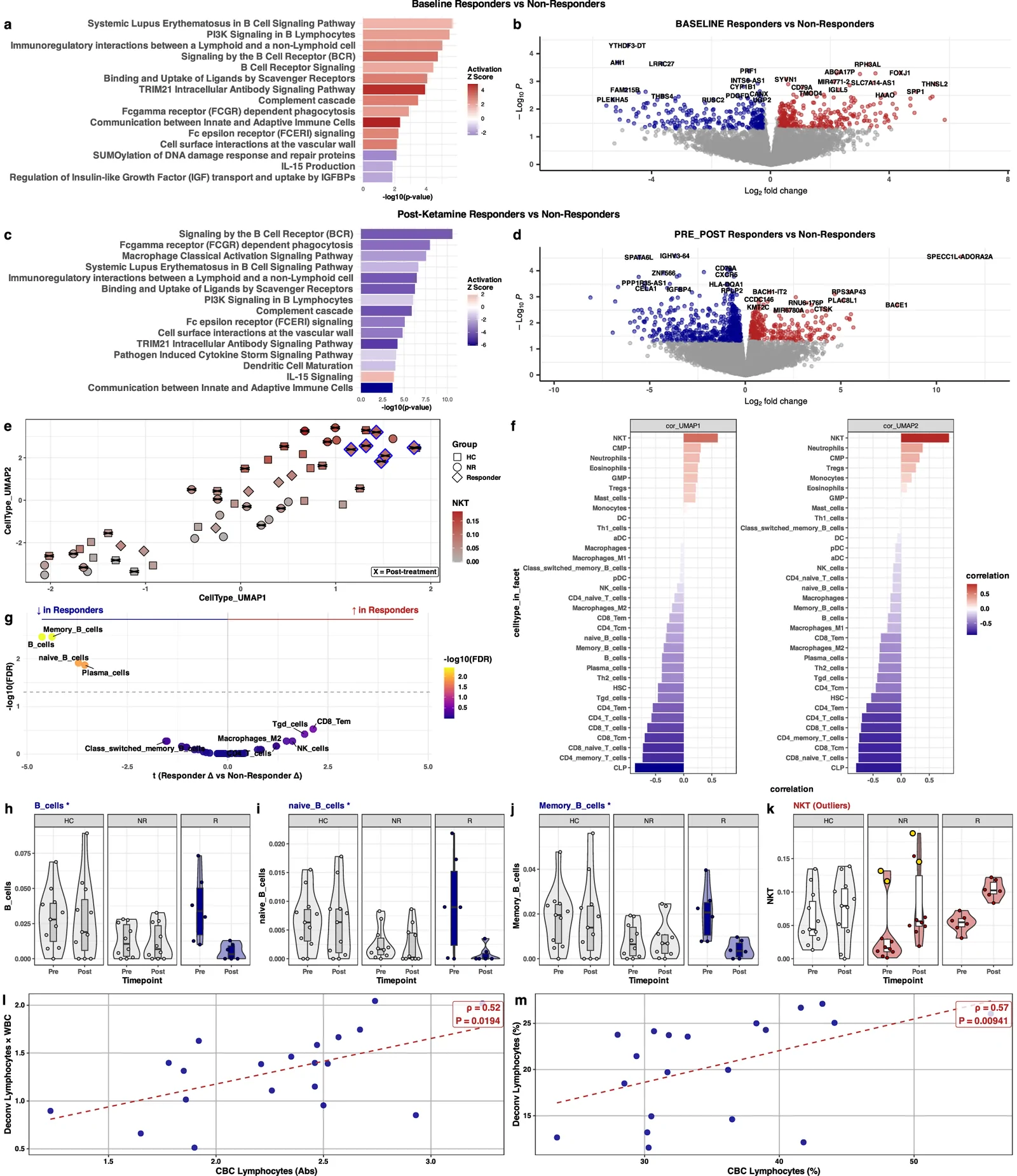
Whole blood transcriptional signatures distinguished ketamine responders (Rs) from non-responders (NRs). **a** Canonical pathways enriched in ketamine Rs versus NRs at baseline identified by Ingenuity Pathway Analysis (IPA). Pathways are ranked by significance (-log10(p-value)), with color intensity representing activation *z*-score (red=positive/activated, blue=negative/inhibited). The top 15 pathways are shown. **b** Volcano plot displaying differentially expressed genes (DEGs) (16,439 genes) between Rs and NRs at baseline. Significant DEGs (p < 0.05; FC > ± 1.2) are highlighted in red (upregulated) or blue (downregulated). Gene labels are shown for the top 50 differentially expressed transcripts by significance. **c, d** Corresponding pathway analysis and volcano plot for treatment effects (pre-post-R versus pre-post-NR comparisons), demonstrating substantial reversal of baseline transcriptional signatures following treatment. **e** Uniform Manifold Approximation and Projection (UMAP) dimensionality reduction plot of cellular composition in individual samples determined by *xCell* deconvolution. Symbol shapes indicate different groupings (healthy volunteers (HVs), NRs, and Rs), and “Xs” demarcate post-treatment timepoints for all groups. Color gradient represents the proportion of natural killer T-cells (NKTs). **f** Ranked correlations between individual immune cell types and UMAP coordinate scores displaying the relative contribution of specific cell populations to natural responder clustering (red=positive correlation, blue=negative correlation). **g** Volcano plot of linear mixed-effects contrasts (R vs NR, pre–post), with significantly reduced B-cell subsets highlighted in blue; annotation arrows indicate directionality of change. **h-k** Violin plots depicting changes in B-cell and NKT proportions between Rs, NRs, and HVs before and after ketamine administration. Blue-colored panels indicate significant decreases in Rs vs NRs (B-cells, naïve B-cells, memory B-cells) for the group × time interaction effects with significant post-hoc contrasts after correction for comparisons across all cell types. Red indicates increases in NKT proportions for Rs and NRs vs HVs but not when comparing R vs NR contrasts; NKT outliers highlighted in gold. **l, m** Correlations between complete blood count (CBC)-derived lymphocyte values and deconvolution estimates at baseline (Spearman’s ρ shown).

After filtration, 16,439 genes were identified. Six hundred and sixteen genes were differentially expressed between ketamine responders (*n* = 6) and non-responders (*n* = 10) at baseline. Post-ketamine, 1061 genes were differentially expressed between responders and non-responders. Eight hundred and ninety-five DEGs (84%) were unique to the post-treatment contrast, whereas 166 genes (16%) were differentially expressed at both timepoints, suggesting most transcriptomic alterations were related to ketamine itself. IPA revealed consistent alterations between groups. At baseline, ketamine responders showed reduced IL‑15 and increased B-cell signaling pathways relative to ketamine non‑responders, along with decreased activation of the insulin secretion signaling pathway, consistent with insulin/IL‑15 signals in our IPSC‑CSF network (Figs. 3a, b). Post-ketamine, IL-15/B-cell alterations largely reversed in responders, though activation in the insulin secretion pathway (*z* = 0.22) did not pass our pre-specified significance threshold (*z* > 1) (Figs. 3c, d). Gene-level evidence, including unaltered apoptosis markers, strong CXCR5 downregulation (an established proxy in peripheral blood for B-cell tissue sequestration [53]), and concordant T-cell activation in other studies [54], favors immune-activation and subsequent lymphoid redistribution over B-cell depletion as an explanation for these findings (see Supplementary Results for details).

Adenosine signaling has recently been implicated as a convergent antidepressant mechanism across ketamine, electroconvulsive therapy, and hypoxia [55]. Here, a read-through transcript for the adenosine A2A receptor (*SPECC1L-ADORA2A*) was the top DEG in whole blood post-ketamine in responders versus non-responders, by a wide margin (Fig. 3d). Legitimate read-through events were confirmed using the Integrative Genomics Viewer (IGV) (Fig. S3a, S4a), validated by multiple alignment methods, and confirmed in publicly available postmortem MDD subgenual anterior cingulate cortex deep sequencing data [56], where expression levels were commensurate with *ADORA2A* itself. In both whole blood and brain, *SPECC1L-ADORA2A* transcripts had ~3-fold longer read lengths than *ADORA2A*, a hallmark of legitimate readthrough transcription (Supplementary Results; Figs. S3,S4).

Our iPSC-CSF network further identified core adenosine mediators: *NT5E* (CD73; extracellular ATP-to-adenosine conversion [55, 57]), *DPP4* (adenosine deaminase complexing protein [58]), and *GPR37* (A2AR surface trafficking regulator [59]). *NT5E* and *DPP4* were very centrally located in network analyses (Figs.2b-d, S2), and this adenosine sub-network was shared only between ketamine (CSF) and psilocybin/LSD, with no differential expression in (2 *R*,6 *R*)-HNK iPSCs (Fig.2c, Table.S2), consistent with recent evidence that (2 *R*,6 *R*)-HNK does not induce adenosine release [55].

Collectively, these findings confirm the peripheral detectability and clinical relevance of core immune and adenosine markers identified in the iPSC-CSF network, further suggesting that immune cell migration/redistribution may be an important, global therapeutic effect of RAADs, consistent with enrichment patterns for cellular adhesion and diapedesis (Fig. 2c-e).

### Cellular correlates of response

Cell-type deconvolution (xCell [41], selected for benchmarked performance on B-cell and IL-15-responsive subsets [60]) revealed that post-treatment samples from ketamine responders naturally grouped together on UMAP clustering (Fig. 3e), driven primarily by NKT cell proportions (Fig. 3f). B-cell types (mature, memory, naïve, plasma) were all significantly reduced post-treatment in ketamine responders versus non-responders after correcting across all 34 cell types (all p_FDR_ < 0.05) (Fig. 3g, j). NKT proportions increased after treatment across all groups; both ketamine responders and non-responders showed significant pre-post increases relative to HVs (post-hoc contrasts p_FDR_ < 0.05) (Fig. 3k) but did not significantly differ by responder status, partly due to outliers (Fig. 3k, **yellow**; Fig. S5).

To further corroborate these findings, CBCs obtained within two weeks of baseline for routine medical screening were examined. Twenty of 27 patients with transcriptomic data had CBCs with accompanying differentials reported. A moderately strong, positive association was observed between CBC-derived lymphocyte fractions and deconvolution-estimated lymphocyte percentages (Spearman’s *ρ* = 0.57, p = 0.009). A similar correlation was observed between CBC-derived absolute lymphocyte counts and patient deconvolution lymphocyte percentage x total white blood cell counts (*ρ* = 0.52, p = 0.019) (Fig. 3l, m).

### Cell-type-specific deconvolution (BayesPrism)

Cell-type-specific deconvolution with *BayesPrism* [44, 61–63] reinforced our initial findings. B-cell subsets showed pathway upregulation at baseline, while NK subsets showed downregulation; both trends reversed post-ketamine in responders versus non-responders (Figs. S6a,S6b). Notably, IL-15 induction of NKs/NKTs is mediated via mTOR signaling [64–66], paralleling RAADs’ effects on synaptic plasticity in the CNS [14, 20]. The NK_3 cluster (expressing canonical NKT markers: *TRDC, KLRB1/CD161, KLRD1/CD94, IL2RB/CD122, KLRC1/NKG2A*) [67,68]) showed the predicted mTOR activation alongside a metabolic shift towards glycolysis/glutaminolysis characteristic of IL-15-activated NKs/NKTs [69,70], supporting peripheral and central mTOR-mediated RAAD effects. For full cell-type-specific results, including the rationale for *BayesPrism* analysis and detailed NK_3 marker characterization, see the discussion accompanying Fig. S6.

### IL-15/IL-7 axis in therapeutic response

The transcriptomic-pathway evidence of IL-15 downregulation and B-cell activation in pre-treatment responders to ketamine suggested that IL-7/IL-15 imbalance may drive pathological immune states in TRD that can differentiate patients by responsiveness to ketamine. IL-7 is the primary driver of human pre-B-cell development [71], and IL-15 can indirectly suppress B-cell overactivity by inducing/activating NKs/NKTs [61, 72–74]. To further assess this hypothesis, MEG-derived gamma power—a leading biomarker of ketamine response and a surrogate for cortical plasticity [32]—was modeled as a function of plasma IL-7 protein concentrations in both TRD and HV participants. Cytokine levels were measured at baseline and 4 h post-infusion (ketamine or placebo); MEG scans were obtained at baseline and 6-9 h post-infusion.

**Fig. 4:**
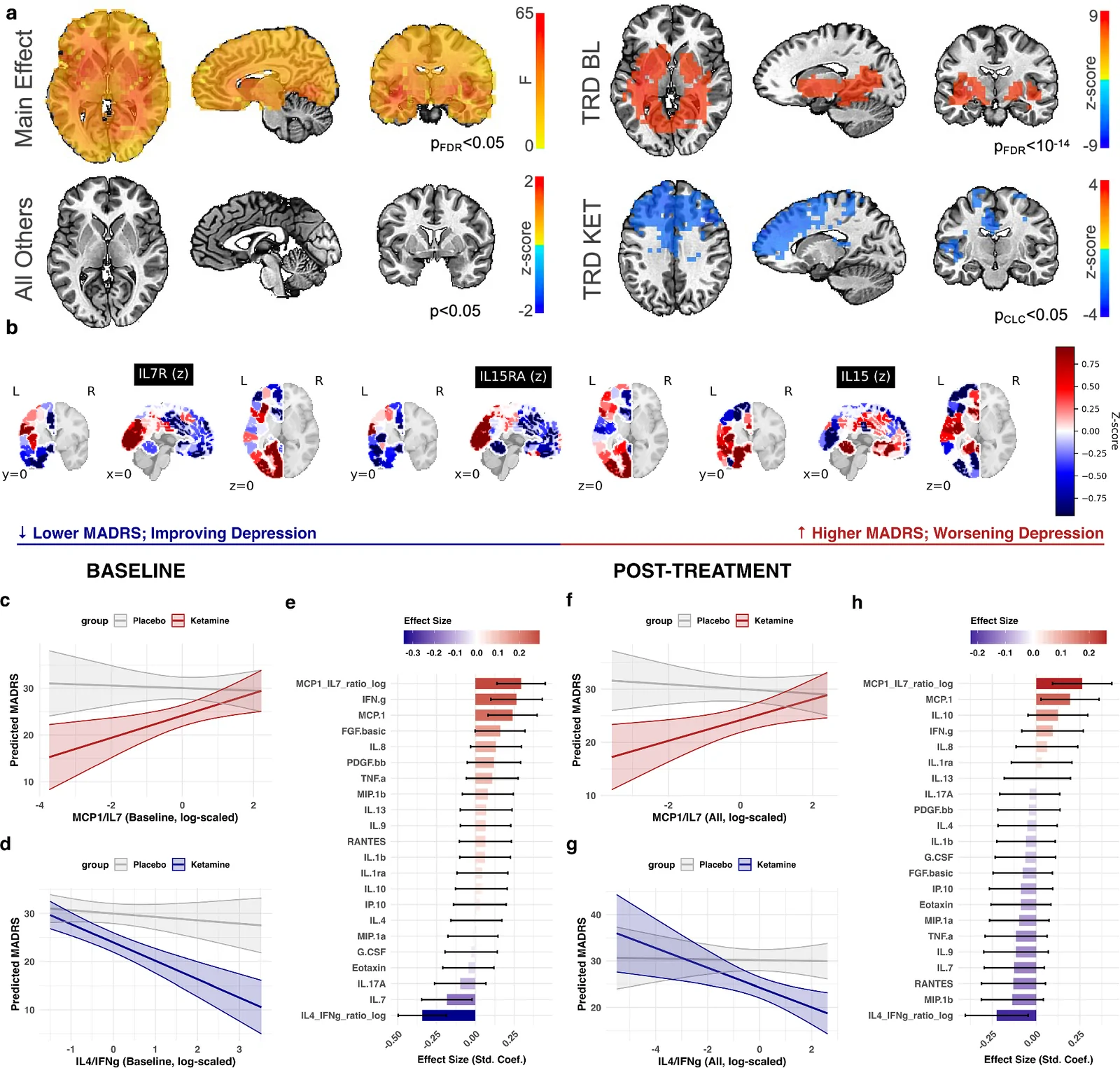
Biomarker trajectories, neuroimaging correlates, and cytokine modulation of antidepressant response to ketamine. **a** Brain source maps revealed magnetoencephalography (MEG)-derived gamma power correlates of interleukin-7 (IL-7) at baseline and post-treatment changes (ketamine and placebo). At baseline, higher plasma IL-7 concentrations were robustly associated with increased gamma power across the brain, particularly in the thalamus, insula, and striatum (strongest effect: q < 10^-14^); this brain-wide relationship was driven entirely by participants with treatment-resistant depression (TRD). Following ketamine treatment, this pattern inverted, as IL-7 levels predicted widespread reductions in cortical gamma power (vertex-wise p_clc_ < 0.05), especially across default mode network (DMN) regions (including the bilateral medial prefrontal cortex, posterior cingulate/precuneus, and anterior cingulate), again at brain-wide significance. **b** Gene expression was measured across the whole brain using microarray data from the Allen Human Brain Atlas (https://human.brain-map.org/). Expression data were mapped to the 268-parcel Shen atlas using the abagen toolbox (https://abagen.readthedocs.io). Processing parameters included ≥2 donors per parcel (i.e., left hemisphere only), 3 mm spatial tolerance, and differential stability probe selection to maximize subcortical representation. Red colors indicate higher expression (*z*-score), while blue represents lower expression levels. **c, d** Predicted Montgomery-Asberg Depression Rating Scale (MADRS) scores illustrating the significant interaction effects between baseline cytokine ratios (monocyte chemoattractant protein 1 (MCP-1)/IL-7(red) and IL-4/interferon-gamma (IFN-γ) (blue)) and treatment condition. Higher baseline MCP-1/IL-7 ratios significantly predicted worse antidepressant response to ketamine (interaction β = 1.59, p_FDR_ < 0.001), while higher baseline IL-4/IFN-γ ratios predicted greater antidepressant response to ketamine (interaction β = −6.23, p_FDR_ < 0.001). **e** Forest plot comparison of standardized effect sizes from linear mixed-effects models assessing baseline cytokine levels as moderators of treatment response (i.e., post-treatment MADRS scores for [Ketamine–Placebo]). Biomarkers with positive effect sizes (red) indicate cytokine levels associated with poorer antidepressant response to ketamine (higher MADRS score), whereas biomarkers with negative effect sizes (blue) indicate positive antidepressant response to ketamine (lower MADRS scores). **f-h** Analogous forest plots to **c-e** for cytokine levels across the full study period (baseline four hours, Day 1, Day 3) (MCP-1/IL-7 interaction β = 2.45, p_FDR_ = 0.009; IL-4/IFN-γ interaction β = −1.67, p_FDR_ = 0.041). Interaction effect p-values are false discovery rate (FDR)-corrected across all four cytokine ratios (baseline and full study; **c, d, f, g**). Effect sizes for individual cytokines are presented as standardized regression coefficients ( ± 95% confidence intervals, using the “effectsize” package in *R*) for comparative purposes, without formal hypothesis testing. All models are adjusted for baseline MADRS score, infusion order, age, sex, body-mass index (BMI) and, additionally, by within-subject average baseline MADRS score before both infusions to address cross-level bias.

At baseline, plasma IL-7 protein levels strongly correlated with higher gamma power, reaching significance across the entire brain (p_fdr_<0.05) (Fig. 4a, **orange**). This relationship was driven entirely by TRD participants and was most robustly (p_fdr_<10^-14^) associated with gamma power in the thalamus, insula, and striatum (Fig.4a, **red**). Post-ketamine, this relationship was inverted, and IL-7 was associated with reduced cortical gamma throughout regions largely encompassing the default mode network (DMN) (i.e., bilateral medial prefrontal cortices, posterior cingulate/precuneus, and portions of the anterior cingulate) (Fig. 4a, **blue**). In contrast to IL-7, IL-15 levels were mostly undetectable ( ~ 80% < LOD) across our plasma multiplex samples—in line with its rapid ( < 1 hour) clearance from plasma after induction [75], predominant cell‑associated presentation (membrane‑bound and/or in IL‑15/IL‑15Rα complexes) [76], and tightly restricted post-transcriptional regulation at rest [77]. Additionally, the IL‑15 LOD (12.82 pg/ml) was the highest among the analytes (mean 1.96 ± 2.99 pg/ml). The only other analyte with LOD > 4 pg/ml (VEGF‑A) was also excluded, suggesting that the 4:1 dilution required for most cytokines may have further limited IL‑15 detection (Fig. S7A). However, assessment of detectable samples (from 18 individuals across various timepoints) showed increasing levels in both TRD and HV participants post-ketamine, in line with our transcriptomic findings (Fig. S7B). Brain-gene expression patterns were plotted for IL-7/IL-15 and their receptors using reference data from the Allen Human Brain Atlas (Fig. 4b) (see Supplement). Baseline IL-7-gamma associations showed some overlap with IL-7 receptor expression patterns, while post-ketamine IL-7-gamma associations more closely resembled brain IL-15 expression patterns as opposed to either receptor.

Notably, IL-15 also regulates MCP-1/CCL-2 expression—another top regulatory hub in our iPSC-CSF overlap network (Fig. 2b-2d)—which has also been associated with peripheral [78] and CSF [79] elevations in depressed patients. Specifically, IL-15-mediated MCP-1 expression in human monocytes is augmented by interferon-gamma (IFN-γ) co-signaling and blocked by IL-4 [80]. Whether ratios of IL-4/IFN-γ and MCP-1/IL-7 predicted response to ketamine was thus also evaluated. As expected, higher baseline MCP-1/IL-7 ratios significantly predicted worse antidepressant response to ketamine versus placebo (interaction β = 1.59, p_FDR_ < 0.001), as did MCP-1/IL-7 ratios across the full study period (interaction β = 2.45, p_FDR_ = 0.009) (Figs. 4c, f). Conversely, higher baseline IL-4/IFN-γ ratios predicted greater antidepressant response to ketamine (interaction β = −6.23, p_FDR_ < 0.001), an effect consistent when considering ratios across the full study (interaction β = −1.67, p_FDR_ = 0.041) (Figs. 4d, g).

To determine the overall importance of these results, standardized effect size estimates for antidepressant outcomes (as assessed via the MADRS) were compared as a function of the interaction between treatment (ketamine versus placebo) and all detectable cytokines (*n* = 20) in our multiplex panel. Separate models for markers at baseline and throughout the study were constructed, similar to those in Figs. 4b-f (see Methods). The core cytokines in our mechanistic framework (i.e., MCP-1, IL-7, IL-4, IFN-γ, and their respective ratios) were consistently the strongest predictors of antidepressant response (and non-response) to ketamine (Figs. 4e, h). Given that MCP-1 appeared to be the strongest predictor of non-response to ketamine, its association with MEG gamma power was subsequently tested; however, no regions survived multiple corrections (Fig. S8). A series of sensitivity analyses run to account for the influence of co-morbid medical diagnoses that could potentially influence immune function and the use of as-needed anti-inflammatory medications during the study period produced largely the same results (Supplementary Results; Figs. S9,S10).

## Discussion

This study identified a convergent molecular signature across pharmacologically distinct RAADs—ketamine, (2 *R*,6 *R*)-HNK, psilocybin, and LSD—characterized by highly similar transcriptional changes in iPSC-derived cortical neurons that aligned with ketamine-induced protein alterations in human CSF (Fig. 2). Network analysis revealed 103 DEPs that formed two functional modules, with a central immune hub (e.g., IL-15, MCP-1/CCL2, CXCL1, CAMs) interconnected to synaptic, insulin, and folate signaling (Fig. 2c, d), suggesting that immune signaling may be a core orchestrator of broader RAAD effects. Critically, these molecular signatures translated to clinical outcomes: peripheral blood transcriptomics from 16 TRD participants revealed that ketamine responders exhibited baseline IL-15 pathway suppression (negative IPA activation z-score) and B-cell activation (positive z-scores) compared to non-responders; both features reversed post-treatment. Plasma IL-7 levels (primary B-cell driver [71]) correlated with MEG-derived gamma power both before and after ketamine, and cytokine ratios relevant to IL-15 signaling [80] (MCP-1/IL-7 and IL-4/IFN-γ) robustly predicted antidepressant efficacy. These findings highlight a potentially conserved neuroimmune mechanism underlying rapid antidepressant effects and provide actionable biomarkers/targets for precision approaches in TRD.

While post-treatment increases in thalamocortical gamma power reliably predict ketamine response [32], prior work from our laboratory demonstrated that baseline gamma power strongly moderates this relationship, with an optimal excitatory range for therapeutic efficacy [81]. Notably, the brain regions where higher baseline gamma most strongly predicted poorer response (thalamus and insula) [81] overlapped almost completely with the strongest IL-7-gamma correlations at baseline here (Fig. 4a), and both relationships were very robust (p_FDR_<10^-14^ for IL-7-gamma; F = 16.92-32.6, p < 0.001 for baseline gamma) [81], suggesting a priority target for validation. Post-ketamine, higher IL-7 levels were associated with gamma reductions within DMN regions.

Although the present study highlights candidate neuroimmune mechanisms, network connectivity changes remain a foundational framework for understanding rapid antidepressant effects. Both ketamine and psilocybin reduce within-DMN hyperconnectivity while increasing between-network connectivity [82–85]. The robust association of IL-7 with gamma power across the brain, its positive relationship with clinical response, and its post-ketamine link to reduced gamma in DMN regions collectively suggest that IL-7 (and, potentially, IL-15) may serve as complementary neuroimmune signals facilitating therapeutic network restructuring and as high-value targets for RAAD optimization, for example with adjunctive immunomodulatory agents [27].

These relationships between cytokine signaling and network connectivity create an apparent paradox. Higher baseline IL-7 was the strongest predictor of clinical improvement, yet IL-7 correlated with higher baseline gamma in regions where gamma is most strongly associated with poor response [81]. This may align with an IL-7/IL-15 rebalancing framework whereby IL-7, within IL-15-deficient conditions, drives pathological neural hyperexcitability and B-cell overactivity. When ketamine restores IL-15, these cytokines may act synergistically to normalize neural and immune activity, potentially through B-cell redistribution. Notably, immune cells treated with equal concentrations of IL-7 and IL-15 display enhanced metabolic flexibility [86,87], most prominently glycolytic capacity [87] and glucose-to-glutamate conversion [86]. While primarily demonstrated in T-cell subpopulations, translational potential exists given the neurotrophic effects of both cytokines [88,89]. Consistent with this, iPSC-derived brain organoids from TRD patients showed elevated oxidative phosphorylation normalized by esketamine through a glycolytic shift [90]—paralleling *BayesPrism* predictions in NK/NKT cells after racemic ketamine (Fig. S6). Notably, arketamine may enhance oxidative phosphorylation instead [91], potentially suggesting that enantiomer-specific metabolic programs may be involved with ketamine.

Despite distinct primary mechanisms—NMDAR antagonism (ketamine), NMDAR-independent action ((2 *R*,6 *R*)-HNK), and 5-HT2A partial agonism (psilocybin, LSD) [92–95]—RAADs converge on glutamate release, α-amino-3-hydroxy-5-methyl-4-isoxazolepropionic acid (AMPA) receptor signaling, and BDNF/mTORC1-dependent plasticity [96–98], consistent with high transcriptional correlation (r = 0.70–0.88; Fig. S1), though our data also revealed meaningful mechanistic divergence.

Specifically, adenosine signaling was recently identified as a conserved antidepressant mechanism [55], and our data offer limited, exploratory human evidence consistent with this possibility. *SPECC1L-ADORA2A* was the top peripheral DEG in ketamine responders by a wide margin, and its existence was also confirmed with multiple alignment methods and in postmortem MDD brain (Fig. S4). Though *SPECC1L-ADORA2A* as a read-through transcript is unlikely to produce functional protein, it may theoretically serve in a regulatory capacity for adenosine signaling via transcriptional interference, chromatin remodeling, or other emerging mechanisms [99–103]. Core adenosine regulators *NT5E* and *DPP4* occupied central positions in the iPSC-CSF overlap networks (Figs. 2b–d, S2), and the adenosine sub-network (including *GPR37*) was shared between ketamine (CSF) and psilocybin/LSD but absent in (2 *R*,6 *R*)-HNK iPSCs (Fig.2c, Table S2), consistent with evidence that (2 *R*,6 *R*)-HNK does not induce adenosine release [55]. Notably, both the adenosine sub-network and IL-15 were shared between ketamine and serotonergic psychedelics but absent with (2 *R*,6 *R*)-HNK (Fig.2c, Table.S2), which may reflect compound-specific mechanistic differences, though substantial heterogeneity across studies limits interpretation. These observations should be regarded as speculative and hypothesis-generating pending direct validation.

Multiple independent lines of evidence support the IL-15/IL-7 framework proposed here. Only two cell types are consistently reduced in depression [104]: NKTs (two studies, 265 patients; SMD = − 0.38) and activated CD16+ NKs (three studies, 110 patients; SMD = − 0.72)—both primarily regulated by IL-15 [62]. In the largest whole-blood transcriptomic mega-analysis in MDD [105], which used the same PBMC single-cell atlas implemented here [44], every single cell population driving the depression‑associated expression profile was either critically IL-15-dependent (NK subsets [NK_1–3]), jointly maintained by IL-7 and IL-15 (CD8 TEM), or strongly IL-15-responsive (CD4_CTLs, γδ T-cells) [62, 106–109], and almost all showed differential expression in responders in our *BayesPrism* simulation (Fig. S6). Additional clinical correlates—including bone metabolism, insulin/adiponectin signaling, and IL-15-mediated MCP-1 release—further support IL-15 pathway involvement and are detailed in the Supplementary Discussion.

Despite these encouraging findings, several limitations warrant consideration. First, the analytical strategy was hypothesis-generating by design; biomarkers were not pre-specified or pre-registered prior to data collection. Unsupervised network approaches (STRING, Cytoscape) were used to identify candidate immune hubs from the iPSC-CSF data. Prioritization of IL-15/MCP-1 pathways that emerged from network topology (degree/betweenness centrality) was based on literature showing that IL-15Rα knockout phenotypes cause depression [73], that IL-15 is a direct and cell-type-specific inducer of MCP-1, and by matching transcriptomic signals in the blood rather than from a priori specification. Other potentially convergent signals, such as adhesion/diapedesis enrichment in the iPSC-CSF network (Fig. 2e) and transcriptomic evidence of B-cell redistribution in whole blood (Fig. 3), point to immune cell migration as a potentially critical mechanism of RAADs, consistent with emerging evidence in psilocybin [110], but could not be pursued further given the methodological constraints of the present datasets (i.e., lacking in vivo cell-based analyses).

Second, the original clinical ketamine trial (NCT00088699) was designed to characterize ketamine’s acute antidepressant effects and collect multimodal phenotyping data to support mechanistic integration of the kind presented here. However, accommodating such diverse modalities introduces temporal alignment limitations. MEG was acquired 6-9 h post-infusion, after ketamine was extensively metabolized and acute dissociative side effects wore off, in order to index plasticity putatively related to AMPA receptor throughput, as opposed to NMDA activity [81, 111]. Blood biomarkers were collected four hours post-infusion for comparability with standard timepoints across other studies [112]. This mismatch represents a potentially suboptimal window for detecting cytokine-MEG relationships. However, convergent evidence from sequential analyses—RNA profiling in ~50% of participants, plasma protein validation in the full cohort with placebo control, and linkage to MEG gamma power across widespread areas (Fig. 4)—suggests these assessment windows were at least sufficiently informative for the key neuroimmune correlates identified here, and less likely to be spurious or detected by chance.

Third, uncorrected *p*-value thresholds were deliberately applied for differential expression and pathway analyses to maximize capture of candidate biological signals. Given that the transcriptomic datasets were intended for exploratory hypothesis generation, the risk of excluding meaningful signals was judged to outweigh the risk of false positives. False discovery was minimized by corroborating significant genes and pathways across independent datasets (CSF, blood, iPSCs, and MEG)—a strategy with established precedent across diverse transcriptomic frameworks [113–118]. All downstream endpoints—CSF proteomics, immune cell type proportions, plasma cytokine ratios with clinical outcomes, and MEG gamma power—underwent appropriate multiple testing corrections.

Fourth, the iPSC-CSF network warrants cautious interpretation on several grounds. Sample sizes were small (*n* = 5 TRD, *n* = 5 HV iPSCs; *n* = 9 HV CSF), iPSC donors were exclusively female, and CSF proteomics were drawn from healthy volunteers only, precluding direct diagnostic comparisons, which were not attempted; all iPSC-CSF findings are presented descriptively. HV and TRD iPSC lines also derived from different somatic sources (fibroblasts and PBMCs, respectively), introducing potential epigenetic memory differences [54]. However, prior work demonstrates high concordance across lines from the same donor regardless of tissue of origin [55]; with reprogramming method accounting for substantially more variance than source tissue [56]. Both cell types were mesodermal in origin and reprogrammed here via the same non-integrating Sendai virus protocol, minimizing lineage bias [57]. The defined Olink panel (*n* = 1460 proteins) captured only a fraction of the transcriptome, and CSF cytokine levels reflect contributions from glia, endothelial cells, and infiltrating immune cells, which are signals potentially distinct from iPSC neuronal responses, and 5-HT2A versus AMPA/NMDA modulation may produce divergent immune effects over time [119,120]. Nevertheless, to our knowledge, this represents the largest published TRD-derived iPSC neuron cohort for RAADs [50], and the only prospective post-ketamine CSF proteomics dataset to date [31]. Six serial CSF draws per participant substantially increase confidence in identified markers. Findings should be treated as candidate signals for conserved signatures, interpreted with appropriate caution, and prioritized for replication in larger, source-matched cohorts.

Despite these limitations, to our knowledge this study is the first to incorporate whole-blood transcriptomics, plasma cytokines, and MEG within the same clinical RAAD cohort, with additional corroboration across CSF proteomics and iPSC-derived neurons. This cross-platform approach enabled identification of convergent immune pathways across central and peripheral compartments, as well as their neurophysiological correlates—providing a comprehensive neurobiological framework that extends beyond single-modality studies. To support transparency, reproducibility, and open science, a self-contained supplementary file with extensively annotated code and preprocessed data is provided, enabling readers to recreate all findings. Given the importance of corroborating RAAD mechanisms in humans [27], all patient-level and biomarker data are also provided and can be additionally connected with MEG and public resting-state fMRI (not analyzed here); see Supplement for instructions.

## Conclusions

This work highlights critical neuroimmune mechanisms that may be shared by ketamine and psychedelic RAADs. Our findings suggest that therapeutic efficacy involves restoring IL-15/IL-7 balance, leading to downstream effects on B-cell homeostasis and neuronal activity. The compound-level specificity demonstrated with adenosine-signaling elements reinforces the potential discriminative utility of our platform and offers timely, convergent human evidence for adenosine as a conserved antidepressant mechanism that may be detectable systemically, though further validation is required. This study broadly identified clinically relevant biomarkers and novel therapeutic targets, underscoring the importance and benefit of multimodal approaches to reverse engineering the mechanism of action underlying RAADs [121].

## Supplementary information

SUPPLEMENTAL MATERIAL

Datasets

## Data Availability

Raw RNA-seq data (iPSC and whole blood) are deposited in the National Center for Biotechnology Information (NCBI) Sequence Read Archive (SRA) (BioProject ID: PRJNA1435237). MEG data will be made publicly available pending additional analyses. For interim access to MEG or other data access inquiries, please contact the corresponding author.

## Code availability

Complete annotated code (R Markdown) and preprocessed data are provided in the Supplementary Data File. Package version control is listed at the end of the Supplement.
