# Supplementary material for: Convergent neuroimmune signaling underlying rapid antidepressant response to ketamine and psychedelics
Source: Mol Psychiatry. 2026 Jul 28;31(12):7251–63. doi: 10.1038/s41380-026-03777-z (PMC13736677; doi:10.1038/s41380-026-03777-z)
Supplement: Supplementary file 1 [file 41380_2026_3777_MOESM1_ESM.docx]

*Jones et al — Convergent Neuroimmune Signaling Underlying Rapid*

*Antidepressant Response to Ketamine and Psychedelics*

**SUPPLEMENT**

**Supplementary Methods**

*Generation of Induced Pluripotent Stem Cell (iPSC)-derived Neurons*

In accordance with National Institutes of Health (NIH) ethical guidelines, peripheral blood mononuclear cells were collected from individuals with treatment-resistant depression (TRD) (n=5) who were enrolled in a crossover trial at the NIH Clinical Center (NCT02484456). The study protocol was approved by the Institutional Review Board, and written informed consent was obtained from all participants prior to any study procedures. Fibroblasts obtained from healthy volunteers (HVs) (n=5) were also collected for reprogramming following standard procurement procedures. All participants were female and ranged in age between 20-68. Additional demographic characteristics can be found in **Supplementary Table S1**.

iPSCs were reprogrammed at the National Heart, Lung, and Blood Institute (NHLBI/NIH) iPSC Core Facility using non-integration (Sendai virus) methods. Lines were authenticated via expression of pluripotency markers and karyotyping, and were consistently confirmed to be mycoplasma-free. The STEMdiff™ embryoid body protocol was followed for 19 days to develop single-cell neural progenitor cells (NPCs). Neural induction was confirmed through visual inspection, and neural rosettes were selected. Cells were seeded onto PLO/laminin-coated dishes (half with poly-L-lysine coated coverslips) at a density of 1.5 x 10^6^ cells/cm^2^. The cells were incubated at 37°C with 5% circulating CO2 and supplemented with BrainPhysTM Neuronal Medium, 1% N2 Supplement-A, 2% Neurocult™ SM1 Neuronal Supplement, 20ng/ml glial-derived neurotrophic factor (GDNF), 20ng/ml brain-derived neurotrophic factor (BDNF), 1mM Dibutyryl-cyclic adenosine monophosphate (cAMP), and 200nM ascorbic acid for 10 weeks with half-medium changes every two days to generate primary cortical neurons. At 10 weeks, iPSC-derived neurons are able to form synapses and have spontaneous synaptic activity alongside other neuronal markers of maturity [1]. All cell lines expressed pluripotency markers and were consistently assessed to be mycoplasma-free. At 10 weeks, the maturity of cortical neurons was confirmed via immunocytochemical staining for MAP2, PSD-95, and β-tubulin III. A CCK-8 cell counting kit was used to determine viability. At 10 weeks maturity, primary cortical neurons were treated with an acetonitrile vehicle (0.1%), (*2R,6R*)-hydroxynorketamine (HNK) (1 uM), ketamine (1 uM), lysergic acid diethylamide (LSD) (10 uM), or psilocybin (10 uM) for 24 hours. Concentrations were chosen based on ranges used in previous research studies [2, 3].

*Bulk RNA-Sequencing of iPSC-Derived Neurons*

For bulk RNA experiments, RNA was immediately isolated following treatment using the Qiagen RNeasy Kit, and RNA purity was confirmed using the NanoDrop spectrophotometer. After isolation, purified RNA was frozen and shipped to Novogene for sample processing. Two x 150 base pair reads were processed on the NovaSeq X Plus Series. FastQ files were downloaded and aligned to human GRCh38 build using STAR [4]. To quantify gene expression, raw counts were generated with featureCounts [5]. Ribosomal and mitochondrial genes were filtered for downstream analysis. Given the differences in cell lineage (fibroblasts and peripheral blood mononuclear cells (PBMCs) from TRD participants and HVs, respectively), diagnosis was conservatively treated as a random effect in data processing (based on recommendations by Hoffman and colleagues [6]) to infer robust conclusions regarding treatment effects across cell lines. Significant contributors to variance (as determined by *variancePartition* [7], sequencing batch and donor), were also included as random effects (0 + ExperimentConditions + (1|Dx) + (1|SequencingBatch) + (1|CellLine)). After removal of minimally expressed genes through edgeR (counts per million > 0.1 in at least five samples), differential analysis for drug (vs vehicle) was conducted with Limma/Dream to handle the mixed effects [6]. Pipeline-specific parameter choices can be found in the relevant sections of the *R* markdown file attached as a supplement (**Supplementary File S2**).

*Clinical Comparison with Cerebrospinal Fluid (CSF) Proteomics*

As an exploratory analysis to determine whether changes observed in the iPSC-derived neurons overlap central changes after ketamine administration in humans, significantly differentially expressed genes (DEGs) from the bulk RNA-seq from our iPSC-derived neurons were compared to CSF proteomics; results from the original experiments have previously been published [8]. Briefly, CSF proteomics were collected at multiple timepoints (40 min, 2 hrs, 4 hrs, 6 hrs, 12 hrs, and 24 hrs) after a single intravenous infusion of racemic ketamine in nine HVs, yielding 54 total CSF samples. Proteomics were performed using the Olink Explore platform (Inflammation I, Oncology I, Cardiometabolic I, and Neurology I reagent kits). We wanted to identify overlapping iPSC targets across the putative rapid-acting agents that would correspond to a “ground-truth” dataset: human, brain-adjacent (CSF) protein shifts following the most clinically established rapid-acting antidepressant (RAAD) (ketamine). We also wanted to distinguish probable drug related effects from inflammatory signals known to be produced by spinal catheters [9].

To avoid falsely eliminating meaningful biological signals during the first stage (see discussion), DEGs from the iPSC conditions were kept at uncorrected (p<0.05), while differential CSF protein expression was false discovery rate (FDR)-corrected for significance(reported in Moaddel and colleagues [8], **Table S5**). For the Olink Explore Platform, the limit of detection (LOD) for individual proteins was not considered in the present study, as it was originally included by the manufacturer for technical evaluation and not to determine biological relevancy. To characterize the biological relevance of conserved mechanisms across agents, a core set of proteins were identified that were differentially expressed across all four rapid-acting antidepressant conditions (ketamine, (2*R*,6*R*)-HNK, LSD, and psilocybin) in iPSC neurons that also showed differential expression in CSF after ketamine administration. These overlapping proteins were then submitted to the STRING database (version 12.0) to identify protein-protein interactions and to construct a functional network. Medium confidence (score > 0.4) was used as the threshold for interaction, with all available interaction sources. The resulting network was imported into Cytoscape (version 3.10.3) for further topological analysis. Network centrality metrics including radiality, undirected edge count, and shortest path length were calculated to identify key hub proteins within the network for plotting. Additionally, for each metric, a *z*-score was calculated to standardize values across the network, enabling comparison of a protein’s centrality (“Betweenness-Centrality”) and connectivity (“Number-Of-Undirected-Edges”) relative to all others. The combined *z*-score (sum of both metric *z*-scores) yielded a holistic measure of network "importance", flagging proteins that are not only highly connected but also serve critical integrative roles. In this context, a high combined *z*-score identifies putative network hubs or connectors that may exert disproportionate control or integration, highlighting them as potentially novel therapeutic or mechanistic targets in depression. This approach allowed us to stratify the network into functional modules and identify potential signaling nodes critical to the rapid antidepressant response mechanism shared across these compounds. For purposes of displaying on the plot (**Fig.2c**), we labeled markers (n=17) that ranked in the top 10 in either combined *z*-score or Number-Of-Undirected-Edges—representing putative top regulators in the two separate hubs identified: the central immune hub (top left of **Fig.2c**) and the peripheral insulin, folate, and plasticity hub (bottom right of **Fig.2c**). Ingenuity pathway analysis (IPA) was run on the entire list of genes (agnostic to expression level between conditions) to probe pathway effects for the RAAD signature (n=103). This was done without expression, as choosing one of the values (either CSF protein or iPSC changes after RAADs) would in theory bias the data towards that drug, undermining our intention to identify shared pathways.

*Clinical Trial of Ketamine in TRD (NCT00088699)*

This was a post-hoc analysis of a previously conducted double-blind, placebo-controlled, randomized, crossover study (NCT00088699) that included 39 participants with TRD and 25 HVs; both groups received a single intravenous infusion of ketamine (0.5 mg/kg) or saline placebo. TRD participants were adults ages 18-65 who fulfilled DSM-IV criteria for major depressive disorder (MDD) without psychotic features as assessed by the Structured Clinical Interview for Axis I DSM-IV Disorders (SCID)-Patient Version and who had not responded to at least two adequate antidepressant trials, as assessed via the Antidepressant Treatment History Form [10]. Patients were experiencing a current depressive episode lasting at least four weeks and were required to have a Montgomery-Åsberg Depression Rating Scale (MADRS) [11] ≥20 at baseline and before each infusion (**Table S3**). Randomization was performed by the National Institutes of Health Clinical Center pharmacy department, all subjects, investigators, and raters were blind to group assignment. Exclusion criteria included drug use (except caffeine/nicotine) within two weeks or a positive toxicology screen (except prescribed benzodiazepines), presence of any medical illness likely to alter brain morphology and/or physiology (e.g.,hypertension, diabetes), even if controlled by medications, abnormal screening laboratory results (listed below), or significant suicidal/homicidal risk (MADRS suicidality score ≥5). All participants were medically evaluated (medical history, physical exam, urine toxicology, urinalysis, ECG, chest x-ray, complete metabolic panel, complete blood count (CBC), thyroid tests, cobalamin (B12), folate (B9) levels) and were deemed in good health. The full study protocol, including all permissible medications (Appendix A) can be accessed at: <https://cdn.clinicaltrials.gov/large-docs/99/NCT00088699/Prot_SAP_000.pdf>. Psychiatric medications (and those prohibited in Appendix A, above) were discontinued ≥2 weeks prior to beginning the study, except aripiprazole (≥3 weeks) and fluoxetine (≥5 weeks). Both TRD and HV participants were admitted to the National Institutes of Health (NIH) Clinical Center for the duration of the study. To control for circadian variation, blood draws and infusions were initiated at approximately equivalent times every morning. Clinical rating scales (MADRS) were administered at baseline, four-hours post-treatment (ketamine or placebo), and then one- and three-days post-treatment.

*Whole Blood Bulk RNA Sequencing (NCT00088699)*

Transcriptomic data from a single sequencing batch (n=11 HV, n=16 TRD) were collected at baseline and four hours post-ketamine to assess early transcriptional changes associated with clinical response (50% MADRS reduction at 24 hours). RNA underwent initial quality control with a NanoDrop™ spectrophotometer (Thermo Fisher Scientific) and an Agilent Bioanalyzer (Agilent Technologies). The mean RNA integrity number (RIN) across samples was 8.2, with a range between 7.5 and 9.2. Stranded library preparation was then performed using the Illumina Globin-Zero protocol (Ribo-Zero Plus kit), designed to remove both rRNA and globin mRNA, enhancing detection of relevant transcripts from whole blood. Sequencing was conducted on an Illumina HiSeq 2500 with a paired-end protocol (100–125 bp), targeting ~60 million reads per sample. Pre- and post-ketamine treatment samples were sequenced within the same flow cell to reduce batch effects in the longitudinal design. FastQ files were adapter-trimmed and assessed for quality with fastP and fastQC respectively [12, 13]. For this part of the analysis, both raw counts and transcripts per million (TPMs) were required to complete differential expression and deconvolution under the same alignment. Reads were thus mapped to the human GRCh38 reference genome using HISAT2 [14] (as opposed to the STAR/featureCounts pipeline above) with Ensembl (version 113) annotations so that raw count matrices and TPMs could be generated simultaneously via StringTie [13] and Ballgown [15] for downstream analysis. Quality assessment was conducted with qualimap [16]. Prior to differential expression analysis, sources of variation in the dataset were assessed using *variancePartition* [7]. Final models incorporated contrasts of interest (response was defined as >50% MADRS reduction at Day 1 and its interaction across pre- and post-ketamine timepoints) while controlling for technical and biological covariates including RNA integrity number (RIN), sex, race (self-identified), and participant as a random effect. Low-abundance genes were filtered using edgeR, retaining only genes with counts per million (CPM) > 0.1 in at least five samples, as above. To accommodate random effects in a repeated measures design, differential expression analysis was performed using Dream [6]. IPA was conducted on DEGs across comparisons [17]. As the primary intent was broad pattern identification that could be corroborated across multiple datasets, significance was set at an uncorrected *p*<0.05 with a fold-change threshold of 1.2 for individual genes. Signaling pathways were filtered by activation *z*-score > 1 and -log(p) >1.3 for each comparison within IPA. Initial cell type deconvolution was implemented using TPMs from the bulk RNAseq alignment via *xCell* (<https://comphealth.ucsf.edu/app/xcell>) [18]. Pipeline-specific parameter choices can be found in the relevant sections of the *R* markdown file attached as a supplement (**Supplementary File S2**).

*Complete blood counts (CBC) Corroboration*

As part of routine medical screening at enrollment, participants underwent CBC testing with differentials. CBC data were retrieved from the medical record and linked to study participants. Of the 27 patients enrolled, 20 had available differential counts suitable for corroboration. For each patient, median values were computed for both relative lymphocyte fraction (%) and absolute lymphocyte count (cells/µL, derived as WBC × lymphocyte%). Because *xCell* deconvolution enrichment scores represent linearized abundance estimates rather than strict proportions, both relative and absolute CBC metrics were calculated from the data to provide complementary corroboration of deconvolution accuracy.

Lymphocyte-like cell fractions from *xCell* deconvolution were calculated based on previously established methods [19]. First, deconvolution “pseudo-percentages” were generated by summing all lymphoid cell type enrichment scores, including B-cells (total, memory, naive, class-switched memory), plasma cells, natural killer (NK) cells, natural killer T-cells (NKTs), CD4+, and CD8+ T-cell subsets (naive, central memory, effector memory), Mucosal-associated invariant T (MAIT) cells, gamma-delta T-cells, Th1/Th2 cells, and regulatory T-cells. Estimated lymphocyte percentages were calculated as 100 × total lymphoid fraction (deconvoluted), while estimated absolute lymphocyte counts were derived as deconvoluted-lymphoid fraction × CBC-derived total white blood cell count. Two separate metrics were calculated to control for the possibility that patients’ total counts could simultaneously drive higher lymphocyte counts on CBCs and greater counts within RNA (for example, if patients were hemoconcentrated due to (de)hydration). Spearman rank correlations were then computed between CBC-measured and deconvolution-estimated lymphocyte values (both percentages and absolute counts).

*Deconvolution with Cell type-specific Differential Expression*

Cell type-specific differential expression analysis was predicted using *BayesPrism* [20]. Hao and colleagues’ multi-omic single-cell PBMC reference dataset (10x Genomics) [21] was used as the reference dataset. It comprised 161,764 PBMCs from eight HVs with simultaneous quantification of single cell RNAseq and cell surface protein abundance via oligonucleotide antibody-derived tags, allowing for high specificity cell-type annotations to inform differential expression priors [21]. Cell-type specific differential expression was modeled under identical parameters as the original bulk RNA data described earlier in this paragraph.

The following parameters were used as recommended by the creators of *BayesPrism* [20]: (1) cell types with <30 cells were excluded; (2) ribosomal/mitochondrial gene groups were excluded, as well as genes expressed in fewer than five cells; (3) deconvolution was performed using only protein coding genes from the reference, to reduce batch effects; and (4) default settings were used for gibbs sampling and outlier removal. Subsequent cell type-specific differential expression analysis was conducted using Dream [6]. As with the original whole blood analysis, final differential expression models incorporated contrasts of interest (response was defined as >50% MADRS reduction at Day 1 and its interaction across pre- and post-ketamine timepoints), while controlling for technical and biological covariates including RIN, sex, race (self-identified), and participant as a random effect.

*Plasma Cytokine Immunoassays*

Plasma cytokine protein levels were quantitated by multiplex immunoassay (Bio-Rad’s Bio-Plex Pro Human Cytokine 27-plex Assay #M500KCAF0Y). Plasma samples were obtained at baseline, four hours, one day, and three days after each infusion. Cytokine assays were performed in duplicate, deidentified, and randomly distributed across plates. The technician was blinded to clinical information and treatment allocation. Analytes included: interleukin (IL)-1b, IL-1ra, IL-2, IL-4, IL-5, IL-6, IL-7, IL-8, IL-9, IL-10, IL-12 (p70), IL-13, IL-15, IL-17A, Eotaxin, fibroblast growth factor (FGF)-basic, granulocyte colony-stimulating factor (G-CSF), granulocyte-macrophage colony-stimulating factor (GM-CSF), interferon gamma (IFN-γ), interferon gamma-induced protein 10 (IP-10/CXCL10), monocyte chemoattractant protein-1 (MCP-1), macrophage inflammatory protein-1 alpha (MIP-1a), platelet-derived growth factor-BB(PDGF-BB), macrophage inflammatory protein-1 beta (MIP-1b), regulated upon activation, normal T-cell expressed and secreted (RANTES/CCL5), tumor necrosis factor alpha (TNF-a), and vascular endothelial growth factor-alpha (VEGF-A). Plasma samples were diluted 1:4 for multiplex cytokines in duplicate blind to clinical information. Levels of all analytes were determined by reading with a Bio-Plex Magpix Multiplex Reader (Bio-Rad, CA). Concentration values were calculated automatically with Bio-Plex Manager MP Software by generating a five-parameter logistic (5-PL) curve-fit standard curve for each analyte. Cytokines with >25% below limit of detection across samples excluded from analyses. The cutoff decision was based on visual inspection of the percentage missing for each analyte, which showed a clear demarcation, with most (n=20) falling approximately below this threshold. For retained cytokines, samples below the level of detection were imputed with the lower limit of detection for each cytokine divided by $\surd2$. Values for individual cytokines, both included and excluded, as well as limit of detection (LOD) and experimental standard data can be found in the Supplementary Data File labeled “FIG4_CYTOKINES.xlsx”. All cytokine values are expressed in pg/ml per manufacturer specification. For excluded cytokines in this file (IL-2, IL-5, IL-6, IL-12(p70), IL-15, GM-CSF, VEGF-A), the combination of the first three variables (Patient_ID, Minutes, and Infusion) can be used to uniquely identify samples and can also be merged with the covariates listed in the “PLASMA_CYTOKINES” tab for those who want to explore these analytes in more detail.

*Association Between Cytokines and Clinical Outcomes*

To evaluate associations between clinical outcomes and plasma cytokines, linear mixed effects models were implemented using the lme4 package in R (version 4.5.0) with the lmerTest extension for Kenward-Roger approximations of degrees of freedom for significance testing. For cytokine analyses, values were log-transformed to address positive skewness and normalized (*z*-scored) prior to modeling to facilitate interpretation across different biomarkers. Because we were primarily concerned with general treatment effects (ketamine versus placebo) for our two hypothesis-driven (from RNAseq) cytokine ratios, we did not have specific hypotheses regarding whether or how treatment effects would vary over time (nor was the model powered to test such effects). Furthermore, as these cytokines operate on different time scales, post-treatment interactions with time would not be feasible or reliable for making consistent cross-cytokine comparisons. To focus power instead on treatment-related effects while controlling for time, post-treatment timepoints were collapsed, controlling for time as a separate fixed effect in an ANCOVA style model. Fixed effects included treatment × marker, time (4 h, Day 1, Day 3), baseline MADRS (just before first infusion), average baseline depression (per infusion; see below), infusion order, age, body-mass index (BMI), and sex; participant was a random intercept. Average baseline depression was calculated as the average of patients’ baseline MADRS score between both infusions and added as an additional covariate based on recommendations by Kenward and Roger, described below [22].

Briefly, in crossover trials, participants are randomized to treatment sequences rather than individual treatments, creating a hierarchical data structure where period-specific measurements are nested within participants. This design feature introduces a unique analytical challenge when incorporating baseline covariates. Standard ANCOVA approaches that use each period-specific baseline as a covariate for its corresponding outcome implicitly assume that within-subject and between-subject regression coefficients are identical—an assumption that is violated except under highly artificial covariance constraints. When random subject effects are used without accounting for this distinction, the resulting treatment effect estimates become biased, a phenomenon known as “cross-level bias” [22]. As demonstrated by Kenward and Roger (2010) [22], this bias is particularly pronounced in orthogonal designs where treatment differences are estimated primarily at the within-subject level, leading to overestimation of baseline adjustment and consequent distortion of treatment effects. To eliminate this bias while retaining the efficiency gains from random effects modeling, Kenward and Roger’s recommended approach of including both the period-specific baseline measurements and the subject-averaged baseline as separate covariates was implemented, allowing the model to estimate distinct regression coefficients for within-subject (period-specific) and between-subject (averaged) baseline effects. This approach preserves the precision advantages of baseline adjustment while ensuring unbiased treatment effect estimates, with simulation studies showing precision improvements equivalent to sample size increases ranging from 3% to 22% compared to conventional methods. Standardized effect sizes for cytokine x treatment interactions were calculated using the Effectsize package in *R* [23].

*Sensitivity Analysis for Inflammatory Comorbidities and Anti-Inflammatory Medications*

Patients enrolled in the clinical crossover trial were excluded if taking regularly scheduled anti-inflammatory medications (e.g., NSAIDs, corticosteroids; see Appendix A for full exclusion criteria: https://cdn.clinicaltrials.gov/large-docs/99/NCT00088699/Prot_SAP_000.pdf). However, intermittent (PRN) analgesic use for minor pain was permitted. Similarly, patients underwent extensive medical screening—confirmed by a medical records review—to exclude comorbidities that could influence brain morphology; certain well-controlled conditions with potential immunological relevance were permitted on a case-by-case basis at the discretion of the treating physician.

Medical diagnoses and medication records were extracted by independent analysts via the NIH Biomedical Translational Research Information System (BTRIS), blinded to study hypotheses. Diagnoses and medications with potential immune relevance were adjudicated by a licensed physician (MD) with immunology training using a separate set of de-identified patient identifiers from those used in statistical analyses. Data were merged and unblinded only after comorbidity classification was complete.

Because patients were admitted at various intervals prior to their first infusion and used varying quantities of different anti-inflammatory agents, several standardization procedures were applied. A peri-infusion lookback window was defined spanning seven days before [24-27] through three days after each infusion (corresponding to the final cytokine timepoint). Within this window, cumulative NSAID exposure was quantified using a validated Assessment of SpondyloArthritis International Society NSAID (ASAS-NSAID) equivalence score, which weights each administered dose relative to the maximum recommended clinical daily dose for that agent [28].

Baseline cytokine levels were compared between NSAID users and non-users and across comorbidity groups (**Fig.S9**). Given the small number of patients with immune-relevant comorbidities (n=4), comorbidity analyses are considered exploratory, and patients were grouped into a single binary flag rather than analyzed by individual diagnosis. Two sets of models were then constructed to assess the influence of these variables on the study's primary findings (**Fig.S10**). First, ASAS score and comorbidity status were each examined as predictors of cytokine levels, including both main effects on baseline levels and interactions with treatment, to determine whether NSAID exposure or immune comorbidity altered cytokine trajectories in response to ketamine versus placebo (**Fig.S10A–B**). Second, the primary cytokine moderation models from **Figure 4** were re-estimated with ASAS score, comorbidity flag, or both entered simultaneously as additional fixed effects, to assess whether these factors confounded the relationship between cytokines and antidepressant response (**Fig.S10C–I**). All models controlled for age, sex, BMI, infusion order, and timepoint.

*Magnetoencephalography (MEG)*

MEG data were drawn from the ketamine crossover trial (NCT00088699). One or two 250-second eyes-closed resting-state MEG datasets were collected at baseline (2-4 days before the first infusion) and 6-9 hours following infusions (post-ketamine and post-placebo). In addition, T1-weighted magnetic resonance imaging scans were collected from each participant in a separate scanning session using a 3T GE scanner and co-registered to the MEG images using MRI-visible fiducial markers placed at the nasion and left and right preauricular locations. During the resting-state recording, participants were instructed to relax with their eyes closed and remain still. Synthetic third gradient balancing was used to attenuate background environment noise. Following acquisition, datasets were high pass filtered at 2 Hz and visually inspected to identify and remove time periods with significant muscular, ocular, or movement artifacts. Up to 10 segments of 15-second clean periods (i.e, no marked artifacts) were identified in an automated fashion, and datasets were discarded if at least five 15-second artifact-free periods could not be identified. Subsequent data analyses used these cleaned datasets.

Synthetic aperture magnetometry (SAM), a linearly constrained minimum-variance beamformer algorithm [29], was used to project source power over the rest period using a 5 mm grid and multisphere head model. Beamformer weights were first calculated using a bandpass frequency of 2-100 Hz, and power was normalized by the projected noise floor of the virtual sensor. The beamformer weights were then applied to the data to compute gamma (30-50 Hz) band power for each voxel over all trials. All images were warped to Talairach space using Analysis of Functional NeuroImages (AFNI) and masked to remove non-brain matter and the cerebellum. The gamma band images were then normalized by the square root of the sum of squared images for the six canonical bands between 2-100 Hz (delta, theta, alpha, beta, gamma, and high gamma). The resulting normalized root-mean-square gamma power images were analyzed using linear mixed-effects models implemented in AFNI (3dLMEr) to test for main effects of IL-7 or monocyte chemoattractant protein-1 (MCP-1). Diagnosis (TRD, HV) and session (baseline, post-placebo, post-ketamine) were included for the individual cytokine models.

*Post-Mortem Brain Gene Expression Analysis*

Whole-brain gene expression data were obtained from the Allen Human Brain Atlas *(https://human.brain-map.org/)*, which provides comprehensive microarray-based expression profiles across six neurotypical adult brains. Expression data were mapped to the 268-parcel Shen atlas using the abagen toolbox *(https://abagen.readthedocs.io)* with parameters optimized for whole-brain coverage including subcortical structures. Processing included: minimum two donors per parcel, 3mm spatial tolerance for sample-to-parcel assignment, differential stability probe selection, and intensity-based filtering (threshold=0.3). Unilateral coverage was chosen given that only two samples in the Allen Human Brain Atlas underwent full bilateral sequencing, after which it was determined that the intra-hemispheric similarity was sufficient for the remainder of the samples to be sequenced unilaterally. Gene expression values were normalized using scaled robust sigmoid normalization, and *z*-scores were calculated across parcels for visualization and analysis. Target genes of interest (IL-7 receptor (IL-7R), IL-15, and IL-15 receptor subunit alpha (IL-15RA)) were identified, and their expression patterns were qualitatively compared with MEG-derived gamma power maps to examine spatial relationships between cytokine receptor distribution and neural oscillatory activity. IL-7 itself did not pass gene quality/filtering thresholds and was thus not displayed.

*Linking Blood-Based Biomarkers from the NIMH Ketamine Mechanism of Action Study (NCT00088699) to Publicly Available Neuroimaging and Additional Covariate and Symptom Inventories*

fMRI data (not analyzed in the current manuscript) from the ketamine crossover trial are publicly available and include a full description of acquisition parameters and cohort characteristics (available at: <doi:10.18112/openneuro.ds005917.v1.0.1>). Individual file names in the repository correspond to the "Patient_ID" identifiers listed in the Excel files accompanying this publication. For example, "sub-MOA101" in the fMRI repository corresponds to the de-identified participant "101M" whose cytokine and RNA-seq biomarker data appear in the attached Excel files. Briefly, fMRI data were collected at baseline ("ses-b0") and at two and 10 days following each infusion. The fMRI repository prefix "ses-d2/d10" denotes post-ketamine scans, while "ses-p2/p10" denotes post-placebo scans. MEG data will be made publicly available upon completion of additional analyses currently under review and will correspond to the 6–9-hour post-infusion timepoint for each condition. De-identified, participant-level nomenclature will be consistent across modalities to facilitate straightforward linkage between blood-based biomarkers and individual MEG scans. For interim access to MEG and other data access inquiries, please contact the corresponding author. Additional symptom inventories and demographic/treatment resistance covariates (summarized in **Table S4**) can be found in Supplementary data file “FIG4_CYTOKINES.xlsx”.

**Supplementary Results**

*B-cell Redistribution Evidence (****Figs.3c,3d****)*

The strong B-cell patterns observed in **Figure 3** may theoretically represent either rapid B-cell depletion or redistribution of activated B-cell populations into lymphoid tissue upon immune activation. Several observations favor the latter. First, though extremely rapid B-cell depletion is possible with targeted therapies [30], ketamine has not demonstrated such effects and lacks a clear mechanism (i.e., anti-CD20) [31]. Second, blood markers of apoptosis (PUMA (BBC3), NOXA (PMAIP1), BAX, CASP3/9/8AP2, BCL2, and PARP1) in the present study were all unaltered (all p>0.18), and nearly all trended towards reduced expression. Third, CXCR5 was also strongly downregulated (t=-4.49, p=0.0001), which is an established proxy in peripheral blood for B-cell tissue sequestration [32] (marker genes extracted from **Figs.3c,3d** recreated here for convenience; also found in the **Fig. 3** data file “TT_R_vs_NR_PRE_POST” tab). Together with transcriptomic signals for acute immune activation (IL-15), corroborated by T-cell studies [31], this suggests that antidepressant response to ketamine may be associated with redistribution of activated immune cell populations as opposed to depletion.

*SPECC1L-ADORA2A Read-Through Transcript Corroboration (****Figs.S3,S4****)*

A recent study found that the antidepressant effects of ketamine, acute intermittent hypoxia, and electroconvulsive therapy all relied on an adenosine surge in the murine prefrontal cortex [33]. A read-through transcript for the A2A receptor (SPECC1L-ADORA2A) was by far the top differentially expressed whole-blood gene post-ketamine in responders versus non-responders (**Fig.3d**). Legitimate read-through events were confirmed in the present study using the Integrative Genomics Viewer (IGV) (**Fig.S3a**), and this transcript persisted with an alternative alignment method (Salmon), where it remained a nominally significant DEG (p=0.07) (**Fig.S3b**)—, consistent with our primary analysis (**Fig. 3d)**. Read lengths averaged ~3-fold greater (7,085 base pairs [bp]) than canonical ADORA2A reads (2,185 bp) (**Fig.S3d**)—a hallmark of true read-through transcription. The presence of SPECC1L-ADORA2A read-through transcripts in the brain was further confirmed using publicly available postmortem subgenual anterior cingulate cortex deep-sequencing data from donors with MDD and controls [34], where normalized expression levels approximated those of ADORA2A itself, again with ~3-fold longer read lengths (**Fig.S4**).

*Extended Description of Findings from BayesPrism Cell-Type Enrichment and Differential Expression (****Fig.S6****)*

B-cells express functional N-methyl-D-aspartate receptors (NMDARs), and non-competitive antagonists such as memantine and ifenprodil inhibit B-cell receptor- and lipopolysaccharide-driven proliferation and immunoglobulin G (IgG) secretion [35], supporting a potential link between B-cell overactivity and our observed ketamine-related transcriptomic effects. Given these established effects, and the strong concordance with our CBC data, underlying cellular activity was examined in greater detail. *BayesPrism* was used with a single cell reference [21] to model immune-cell-type-specific differential expression patterns [36-38]. Forty-eight cell types with sufficiently distinct marker DEGs (see [20]) were included in this analysis (**Table S5).** Instead of pathway-specific IPA, competitive, rank-based enrichment (via Dream/Zenith [6, 39]) was used here to broadly assess functional changes across predicted cell types (with Gene Ontology Molecular Function (GO:MF) terms). This conservative approach was intended to provide a more unbiased overview of altered cellular activity, which minimizes sensitivity to artificial expression differences that may arise from *BayesPrism’s* use of single-cell reference priors [40].

NK-relevant findings can be found in the main text. For B-cell populations at baseline, ketamine responders exhibited overactivity in memory and naive lambda B-cell subsets, characterized by markers such as *IGLC2, IGLC3*, and *TCL1A*, while showing underactivity in intermediate lambda B-cells, which expressed CD22 (*SIGLEC-2*) as a key differentiating marker. Following ketamine treatment, significant downregulation was observed across virtually all B-cell populations, including both kappa and lambda variants of naive, memory, and intermediate subsets. This broad suppressive effect on B-cell activity post-treatment aligns with our differential expression and prior deconvolution data, suggesting a comprehensive modulation of humoral immunity in ketamine responders.

In the T-cell compartment, the CD4+ effector memory T-cell subtype (CD4 TEM_2) is the only CD4 TEM subset (of four) that does not express *IL-7R* as a marker gene and is also predicted to have the most differential activity in ketamine responders (baseline and after treatment). Its markers indicate robust effector functions, including *GZMK* (cytotoxicity), TIGIT (inhibitory receptor), *IFNG-AS1* (IFN-γ regulation), and *CD40LG* (T-cell help). The upregulation of pathways related to antigen recognition, T-cell receptor signaling, and protein synthesis, coupled with downregulation of calmodulin and phosphatidylinositol-3,4,5-trisphosphate binding, indicates a cell state primed for rapid effector responses. This subtype appears to be highly responsive to antigenic stimuli and poised for immediate effector functions, potentially representing a more terminally differentiated or recently activated state within the CD4 TEM compartment. Post-ketamine, this primed effector type is predicted to be downregulated in responders.

At baseline, ketamine responders also exhibited a distinct CD8+ T-cell profile characterized by increased activity of naïve cells and decreased representation of specific memory subsets (TCM_2, TEM_3, and TEM_5). The downregulated TCM_2 population is distinguished by IL-7R and GATA3 expression, suggesting a homeostatic memory subset dependent on IL-7 signaling, while the diminished TEM_3 and TEM_5 populations display cytotoxic profiles (GZMK/KLRD1 and GZMH/GNLY/GZMB, respectively) with features of terminally differentiated effector cells. Following ketamine treatment, expansion of TEM_1 and TEM_4 subsets, which share cytotoxic markers but exhibit distinct cytokine and activation profiles, was observed. This pattern aligned with our IL-7/IL-15 rebalancing hypothesis, as the expanded post-treatment subsets lacked *IL-7R* expression but had markers consistent with IL-15 responsiveness, especially NK-cell associated receptors (NKG7, KLRD1). The shift from IL-7R-expressing central memory cells toward terminally differentiated effector populations suggests that ketamine may promote CD8+ T-cell differentiation through preferential IL-15 signaling, potentially enhancing cytotoxic function while reducing dependence on IL-7-mediated homeostasis.

*Sensitivity Analysis for The Influence of Comorbid Medical Illnesses and Anti-Inflammatory Medication (****Figs.S9,S10****)*

Several sensitivity analyses were conducted to assess whether comorbid inflammatory conditions or anti-inflammatory medication use could bias cytokine findings. Although patients taking scheduled NSAIDs or corticosteroids were excluded at screening, as-needed (PRN) use was permitted. A validated ASAS-NSAID equivalence score was calculated for each patient over a peri-infusion window spanning seven days before [24-27] through three days after each infusion, quantifying cumulative NSAID exposure relative to the maximum recommended daily dose [28]. Approximately half of TRD patients used PRN NSAIDs during this period (n=18–22/39 across infusions; **Fig.S9a**), consistent with pooled prevalence estimates of comorbid pain in depression (~40–60%) [41, 42].

Regarding medical comorbidities, patients were extensively screened and excluded for conditions that could influence brain morphology. Four patients had autoimmune or allergic diagnoses judged by a blinded physician to potentially influence immune function (allergic rhinitis n=2, celiac disease n=1, Hashimoto's thyroiditis n=1). All had C-reactive protein (CRP) levels <2 mg/L at baseline and throughout the study. The patient with Hashimoto's thyroiditis had been stable on thyroid replacement for years prior to enrollment, with thyroid-stimulating hormone (TSH) levels remaining within normal limits across all study timepoints.

Given the small number of patients with immune comorbidities (n=4), all comorbidity analyses should be considered exploratory. Cell sizes were insufficient to support statistical comparisons across the full 2×2 combination of NSAID use and comorbidity status; NSAID exposure was therefore examined as the primary grouping variable, with comorbid patients highlighted to allow visual inspection of their distribution (**Fig.S9b**). Baseline cytokine levels were largely unaffected by NSAID use (all p-uncorrected>0.05), with the exception of IL-4 (p=0.018) and Eotaxin (p=0.049), neither of which survived Benjamini-Hochberg correction (p-adj=0.36 and p-adj=0.49, respectively; **Fig.S9b**), consistent with the covariate-adjusted primary models (**Fig.4**).

The study also examined whether NSAID exposure or comorbidity status could confound cytokine responses to treatment or cytokine moderation of antidepressant response. First, ASAS score and comorbid illness were each entered separately as covariates in models predicting cytokine levels, assessing both the main effects on baseline levels and interactions with treatment (**Figs.S10a,S10b**). Across these models, the only significant effect was a greater ketamine-induced reduction in IL-1ra among patients with higher NSAID exposure (p=0.001, **Fig.S10a**), consistent with prior work using NSAIDs alongside experimental immune stimulation [43, 44]. Second, to assess whether these variables could confound cytokine moderation of antidepressant response, ASAS score and comorbidity were entered separately (**Fig.S10c-f**) and jointly (**Fig.S10g-i**) as covariates in the primary cytokine × treatment interaction models predicting MADRS score. Cytokine × treatment interactions from **Figure 4** were unchanged across all model specifications, and fixed effect estimates for ASAS score and comorbidity were uniformly non-significant, indicating that PRN NSAID use and autoimmune/allergic diagnoses did not confound the primary findings.

**Supplementary Tables**

**Supplementary Table S1: Differential Gene Expression in iPSC-Derived Neurons Following Treatment with Rapid-Acting Antidepressants** The top portion of the table summarizes the number of DEGs in iPSC-derived neurons following 24-hour exposure to various rapid-acting antidepressant compounds. Cell lines were derived from female patients with TRD (n=5, PBMC-derived) and HVs (n=5, fibroblast-derived). All treatments were compared against 0.1% acetonitrile vehicle control, with significance threshold set at p < 0.05. The psychedelic compounds (LSD and psilocybin) induced more extensive transcriptional changes than ketamine or its metabolite (2*R*,6*R*)-HNK**.** Fewer ketamine samples were sequenced due to several having unexpectedly low viability compared to all other samples at the time of RNA processing.

| **Cell Lines** | **Source** | **Treatments (24hrs)** | **Concentrations** | **TRD Samples** | **HV Samples** | **Number of DEGs** *(vs Acetonitrile 0.1% Vehicle)* |
| --- | --- | --- | --- | --- | --- | --- |
| **TRD** (n = 5)  All female  PBMC-derived | | (2*R*,6*R*)-HNK | 1 uM | 7 | 11 | 802 |
|  |  | Ketamine | 1 uM | 4 | 1 | 310 |
| **HV** (n = 5)  All female  Fibroblast-derived | | Psilocybin | 10 uM | 7 | 4 | 1450 |
|  |  | LSD | 10 uM | 6 | 5 | 1883 |
|  |  | Vehicle | *Acetonitrile 0.1%* | 5 | 7 | N/A |

Abbreviations: iPSC: induced pluripotent stem cells; TRD: treatment-resistant depression; HV: healthy volunteer; DEGs: differentially expressed genes; (2*R*,6*R*)-HNK: (2R,6*R*)-hydroxynorketamine; PBMCs: peripheral blood mononuclear cells

**Supplementary Table S2.** **Overlap between induced pluripotent stem cell (iPSC)-derived gene expression signatures and cerebrospinal fluid (CSF) proteomic results across treatment conditions.** This table summarizes genes identified in both the iPSC differential expression results and in CSF proteomic analyses across timepoints. The “iPSC Conditions & CSF Timepoints” column lists the specific treatment conditions and CSF collection timepoints in which each gene was found. The “Drugs” column reports the unique treatments ((2*R*,6*R*-hydroxynorketamine (HNK), LSD, psilocybin, ketamine) associated with each gene, while “Num Drugs” provides a count of how many distinct drugs were associated with each gene. Num Pairs indicates how many CSF-timepoint x Drug combinations there were. Agree_pct indicates what proportion of the IPSC-CSF pairings for that gene/protein were expressed in the same direction (up- vs down-regulated) at a given CSF timepoint. ipsc_dir and csf_dir indicate the direction of expression (values of 100 and 0 indicate that for every CSF timepoint the direction of expression remained consistent across all measures).

| Gene/Protein | iPSC Conditions & CSF Timepoints  (Formatted as “iPSC Condition_CSF Draw Time”  (minutes post-ketamine infusion)) | Drugs | Num Drugs | n_pairs | agree_pct | ipsc_dir | csf_dir |
| --- | --- | --- | --- | --- | --- | --- | --- |
| ACVRL1 | HNK124_1440; LSD24_1440; Psil1024_1440 | HNK; LSD; Psil | 3 | 3 | 100 | Up | Up |
| CCL2 | HNK124_1440; HNK124_230; HNK124_360; HNK124_720; LSD24_1440; LSD24_230; LSD24_360; LSD24_720; Psil1024_1440; Psil1024_230; Psil1024_360; Psil1024_720 | HNK; LSD; Psil | 3 | 12 | 100 | Up | Up |
| CCN3 | HNK124_1440; HNK124_230; HNK124_360; HNK124_720; LSD24_1440; LSD24_230; LSD24_360; LSD24_720; Psil1024_1440; Psil1024_230; Psil1024_360; Psil1024_720 | HNK; LSD; Psil | 3 | 12 | 100 | Up | Up |
| CD74 | HNK124_1440; HNK124_360; HNK124_720; LSD24_1440; LSD24_360; LSD24_720; Psil1024_1440; Psil1024_360; Psil1024_720 | HNK; LSD; Psil | 3 | 9 | 100 | Up | Up |
| CHI3L1 | HNK124_1440; HNK124_360; HNK124_720; LSD24_1440; LSD24_360; LSD24_720; Psil1024_1440; Psil1024_360; Psil1024_720 | HNK; LSD; Psil | 3 | 9 | 100 | Up | Up |
| CTSS | HNK124_1440; LSD24_1440; Psil1024_1440 | HNK; LSD; Psil | 3 | 3 | 100 | Up | Up |
| CXCL1 | HNK124_120; HNK124_230; HNK124_360; HNK124_720; LSD24_120; LSD24_230; LSD24_360; LSD24_720; Psil1024_120; Psil1024_230; Psil1024_360; Psil1024_720 | HNK; LSD; Psil | 3 | 12 | 100 | Up | Up |
| ICAM1 | HNK124_1440; LSD24_1440; Psil1024_1440 | HNK; LSD; Psil | 3 | 3 | 100 | Up | Up |
| ICOSLG | HNK124_1440; HNK124_230; HNK124_360; HNK124_720; LSD24_1440; LSD24_230; LSD24_360; LSD24_720; Psil1024_1440; Psil1024_230; Psil1024_360; Psil1024_720 | HNK; LSD; Psil | 3 | 12 | 100 | Up | Up |
| IL1R1 | HNK124_1440; LSD24_1440; Psil1024_1440 | HNK; LSD; Psil | 3 | 3 | 100 | Up | Up |
| LGALS3 | HNK124_230; LSD24_230; Psil1024_230 | HNK; LSD; Psil | 3 | 3 | 100 | Up | Up |
| MCAM | HNK124_1440; LSD24_1440; Psil1024_1440 | HNK; LSD; Psil | 3 | 3 | 100 | Up | Up |
| PLAT | HNK124_720; LSD24_720; Psil1024_720 | HNK; LSD; Psil | 3 | 3 | 100 | Up | Up |
| SDC4 | HNK124_1440; HNK124_720; LSD24_1440; LSD24_720; Psil1024_1440; Psil1024_720 | HNK; LSD; Psil | 3 | 6 | 100 | Up | Up |
| BMP6 | LSD24_1440; LSD24_360; LSD24_720; Psil1024_1440; Psil1024_360; Psil1024_720 | LSD; Psil | 2 | 6 | 0 | Down | Up |
| C2 | LSD24_1440; Psil1024_1440 | LSD; Psil | 2 | 2 | 100 | Up | Up |
| CD109 | LSD24_1440; LSD24_230; LSD24_360; LSD24_720; Psil1024_1440; Psil1024_230; Psil1024_360; Psil1024_720 | LSD; Psil | 2 | 8 | 100 | Up | Up |
| CPM | HNK124_1440; HNK124_360; HNK124_720; LSD24_1440; LSD24_360; LSD24_720 | HNK; LSD | 2 | 6 | 100 | Up | Up |
| CPPED1 | LSD24_120; LSD24_1440; LSD24_230; LSD24_360; LSD24_720; Psil1024_120; Psil1024_1440; Psil1024_230; Psil1024_360; Psil1024_720 | LSD; Psil | 2 | 10 | 100 | Up | Up |
| DKK1 | LSD24_1440; LSD24_720; Psil1024_1440; Psil1024_720 | LSD; Psil | 2 | 4 | 100 | Up | Up |
| DPP4 | LSD24_1440; Psil1024_1440 | LSD; Psil | 2 | 2 | 100 | Up | Up |
| DSG2 | LSD24_1440; LSD24_360; Psil1024_1440; Psil1024_360 | LSD; Psil | 2 | 4 | 100 | Up | Up |
| GPR37 | LSD24_1440; LSD24_230; LSD24_720; Psil1024_1440; Psil1024_230; Psil1024_720 | LSD; Psil | 2 | 6 | 100 | Up | Up |
| GRN | HNK124_1440; LSD24_1440 | HNK; LSD | 2 | 2 | 100 | Up | Up |
| IFNGR2 | LSD24_1440; LSD24_230; LSD24_360; LSD24_720; Psil1024_1440; Psil1024_230; Psil1024_360; Psil1024_720 | LSD; Psil | 2 | 8 | 100 | Up | Up |
| IL15 | LSD24_1440; LSD24_230; LSD24_720; Psil1024_1440; Psil1024_230; Psil1024_720 | LSD; Psil | 2 | 6 | 100 | Up | Up |
| IL18R1 | LSD24_1440; LSD24_230; LSD24_720; Psil1024_1440; Psil1024_230; Psil1024_720 | LSD; Psil | 2 | 6 | 100 | Up | Up |
| LGALS8 | LSD24_1440; LSD24_230; LSD24_360; LSD24_720; Psil1024_1440; Psil1024_230; Psil1024_360; Psil1024_720 | LSD; Psil | 2 | 8 | 100 | Up | Up |
| LIF | HNK124_120; HNK124_1440; HNK124_230; HNK124_360; HNK124_720; Psil1024_120; Psil1024_1440; Psil1024_230; Psil1024_360; Psil1024_720 | HNK; Psil | 2 | 10 | 100 | Up | Up |
| MATN3 | LSD24_1440; LSD24_720; Psil1024_1440; Psil1024_720 | LSD; Psil | 2 | 4 | 100 | Up | Up |
| NECTIN2 | HNK124_1440; HNK124_720; Psil1024_1440; Psil1024_720 | HNK; Psil | 2 | 4 | 100 | Up | Up |
| NMNAT1 | HNK124_230; LSD24_230 | HNK; LSD | 2 | 2 | 100 | Up | Up |
| OSMR | LSD24_1440; Psil1024_1440 | LSD; Psil | 2 | 2 | 100 | Up | Up |
| SIRPB1 | HNK124_1440; HNK124_720; Psil1024_1440; Psil1024_720 | HNK; Psil | 2 | 4 | 100 | Up | Up |
| PTGDS | LSD24_1440; LSD24_720; Psil1024_1440; Psil1024_720 | LSD; Psil | 2 | 4 | 0 | Up | Down |
| QPCT | LSD24_1440; LSD24_360; LSD24_720; Psil1024_1440; Psil1024_360; Psil1024_720 | LSD; Psil | 2 | 6 | 100 | Up | Up |
| SHMT1 | HNK124_120; HNK124_230; LSD24_120; LSD24_230 | HNK; LSD | 2 | 4 | 100 | Up | Up |
| SIRPB1 | HNK124_1440; LSD24_1440 | HNK; LSD | 2 | 2 | 0 | Down | Up |
| TFPI2 | LSD24_1440; LSD24_230; LSD24_360; LSD24_720; Psil1024_1440; Psil1024_230; Psil1024_360; Psil1024_720 | LSD; Psil | 2 | 8 | 100 | Up | Up |
| TNFRSF13C | LSD24_1440; LSD24_230; LSD24_360; LSD24_720; Psil1024_1440; Psil1024_230; Psil1024_360; Psil1024_720 | LSD; Psil | 2 | 8 | 100 | Up | Up |
| ACAN | LSD24_1440; LSD24_720 | LSD | 1 | 2 | 0 | Down | Up |
| ACP5 | KET24_1440; KET24_360; KET24_720 | KET | 1 | 3 | 100 | Up | Up |
| ADGRB3 | LSD24_1440; LSD24_230; LSD24_360; LSD24_720 | LSD | 1 | 4 | 0 | Down | Up |
| ADGRE2 | Psil1024_1440; Psil1024_720 | Psil | 1 | 2 | 100 | Up | Up |
| ADGRG1 | LSD24_720 | LSD | 1 | 1 | 0 | Down | Up |
| BST2 | LSD24_230 | LSD | 1 | 1 | 100 | Up | Up |
| CA2 | Psil1024_120 | Psil | 1 | 1 | 100 | Up | Up |
| CD164 | LSD24_1440; LSD24_360; LSD24_720 | LSD | 1 | 3 | 100 | Up | Up |
| CD276 | HNK124_1440; HNK124_360; HNK124_720 | HNK | 1 | 3 | 100 | Up | Up |
| CDCP1 | Psil1024_120; Psil1024_1440; Psil1024_230; Psil1024_360 | Psil | 1 | 4 | 100 | Up | Up |
| CLMP | HNK124_1440 | HNK | 1 | 1 | 100 | Up | Up |
| CNDP1 | LSD24_1440; LSD24_360; LSD24_720 | LSD | 1 | 3 | 0 | Down | Up |
| CNTNAP2 | LSD24_1440; LSD24_230; LSD24_360; LSD24_720 | LSD | 1 | 4 | 0 | Down | Up |
| CPB1 | LSD24_120; LSD24_1440; LSD24_230; LSD24_360; LSD24_720 | LSD | 1 | 5 | 100 | Up | Up |
| CPXM1 | LSD24_720 | LSD | 1 | 1 | 100 | Up | Up |
| CRLF1 | Psil1024_1440; Psil1024_720 | Psil | 1 | 2 | 0 | Down | Up |
| CXCL16 | LSD24_1440; LSD24_360; LSD24_720 | LSD | 1 | 3 | 100 | Up | Up |
| DBI | LSD24_1440; LSD24_360; LSD24_720 | LSD | 1 | 3 | 0 | Down | Up |
| DRAXIN | LSD24_1440; LSD24_230; LSD24_360; LSD24_720 | LSD | 1 | 4 | 0 | Down | Up |
| EFNA1 | Psil1024_1440; Psil1024_360 | Psil | 1 | 2 | 100 | Up | Up |
| EPHB4 | HNK124_1440; HNK124_230; HNK124_360; HNK124_720 | HNK | 1 | 4 | 100 | Up | Up |
| ESM1 | Psil1024_1440; Psil1024_720 | Psil | 1 | 2 | 0 | Down | Up |
| F3 | LSD24_1440; LSD24_230; LSD24_360; LSD24_720 | LSD | 1 | 4 | 0 | Down | Up |
| FCGR2A | KET24_1440; KET24_720 | KET | 1 | 2 | 0 | Down | Up |
| FKBP4 | LSD24_120; LSD24_1440; LSD24_230; LSD24_360; LSD24_720 | LSD | 1 | 5 | 0 | Down | Up |
| FLT1 | Psil1024_1440 | Psil | 1 | 1 | 0 | Down | Up |
| FLT3LG | HNK124_1440; HNK124_230; HNK124_360; HNK124_720 | HNK | 1 | 4 | 0 | Down | Up |
| FUCA1 | Psil1024_120; Psil1024_1440; Psil1024_230; Psil1024_360; Psil1024_720 | Psil | 1 | 5 | 100 | Up | Up |
| FYB1 | LSD24_120; LSD24_230 | LSD | 1 | 2 | 0 | Down | Up |
| GLRX | Psil1024_230; Psil1024_720 | Psil | 1 | 2 | 100 | Up | Up |
| HMBS | Psil1024_120 | Psil | 1 | 1 | 100 | Up | Up |
| HMOX1 | LSD24_1440; LSD24_230; LSD24_720 | LSD | 1 | 3 | 100 | Up | Up |
| ICAM5 | LSD24_360; LSD24_720 | LSD | 1 | 2 | 0 | Down | Up |
| IGFBP1 | LSD24_1440; LSD24_720 | LSD | 1 | 2 | 100 | Up | Up |
| IGFBP4 | LSD24_1440 | LSD | 1 | 1 | 100 | Up | Up |
| IL1RAP | Psil1024_1440 | Psil | 1 | 1 | 100 | Up | Up |
| IL1RL2 | HNK124_1440; HNK124_230; HNK124_360; HNK124_720 | HNK | 1 | 4 | 100 | Up | Up |
| MET | HNK124_1440; HNK124_360; HNK124_720 | HNK | 1 | 3 | 100 | Up | Up |
| MMP3 | HNK124_1440; HNK124_360; HNK124_720 | HNK | 1 | 3 | 100 | Up | Up |
| NCAM1 | LSD24_1440; LSD24_230; LSD24_360; LSD24_720 | LSD | 1 | 4 | 0 | Down | Up |
| NOMO1 | Psil1024_1440 | Psil | 1 | 1 | 100 | Up | Up |
| NRCAM | Psil1024_120; Psil1024_1440; Psil1024_230; Psil1024_360; Psil1024_720 | Psil | 1 | 5 | 0 | Up | Down |
| NT5E | Psil1024_1440; Psil1024_230 | Psil | 1 | 2 | 100 | Up | Up |
| PECAM1 | LSD24_1440 | LSD | 1 | 1 | 100 | Up | Up |
| PLIN3 | HNK124_230; HNK124_720 | HNK | 1 | 2 | 100 | Up | Up |
| PTS | HNK124_1440; HNK124_230 | HNK | 1 | 2 | 0 | Down | Up |
| ROBO1 | LSD24_1440; LSD24_720 | LSD | 1 | 2 | 0 | Down | Up |
| ROBO2 | LSD24_1440; LSD24_230; LSD24_360; LSD24_720 | LSD | 1 | 4 | 0 | Down | Up |
| ROR1 | HNK124_1440; HNK124_720 | HNK | 1 | 2 | 100 | Up | Up |
| SCG3 | LSD24_360; LSD24_720 | LSD | 1 | 2 | 100 | Down | Down |
| SERPINB1 | LSD24_1440; LSD24_720 | LSD | 1 | 2 | 100 | Up | Up |
| SERPINB6 | LSD24_1440; LSD24_360; LSD24_720 | LSD | 1 | 3 | 100 | Up | Up |
| SIGLEC10 | LSD24_720 | LSD | 1 | 1 | 0 | Down | Up |
| SIRPA | Psil1024_1440; Psil1024_230; Psil1024_360; Psil1024_720 | Psil | 1 | 4 | 0 | Down | Up |
| SLITRK6 | HNK124_1440 | HNK | 1 | 1 | 100 | Up | Up |
| SOD2 | LSD24_1440; LSD24_230; LSD24_360; LSD24_720 | LSD | 1 | 4 | 100 | Up | Up |
| TEK | LSD24_1440; LSD24_230; LSD24_360; LSD24_720 | LSD | 1 | 4 | 0 | Down | Up |
| THBS2 | LSD24_1440; LSD24_720 | LSD | 1 | 2 | 100 | Up | Up |
| TNFRSF12A | Psil1024_1440; Psil1024_720 | Psil | 1 | 2 | 100 | Up | Up |
| TNFRSF14 | Psil1024_1440; Psil1024_230; Psil1024_360; Psil1024_720 | Psil | 1 | 4 | 100 | Up | Up |
| TNFSF14 | LSD24_720 | LSD | 1 | 1 | 100 | Up | Up |
| VCAM1 | HNK124_1440; HNK124_720 | HNK | 1 | 2 | 100 | Up | Up |
| VWA1 | LSD24_1440; LSD24_230; LSD24_720 | LSD | 1 | 3 | 100 | Up | Up |

**Supplementary** **Table S3: Baseline demographic and clinical characteristics of study participants.** Demographic and clinical variables for HVs (n=25) and patients with TRD (n=39) enrolled in a randomized, double-blind, placebo-controlled crossover trial of intravenous ketamine **(NCT00088699).** The TRD cohort represents a highly treatment-refractory population, with patients having failed to respond to a mean of 4.95 adequate antidepressant trials and 3.32 augmentation strategies, as defined by the Antidepressant Treatment History Form (ATHF) established by Sackeim and colleagues [10]. Notable characteristics include high rates of unemployment (84.6%), prior ECT treatment (25.6%), lithium augmentation failure (41.0%), and trauma history (46.2%). The negligible placebo response rates (0% for ≥50% MADRS reduction; 2.8% for ≥25% reduction) underscore the severity and treatment resistance of this sample. Values are presented as mean (standard deviation) for continuous variables and n (%) for categorical variables. Lithium augmentation defined as > 900 mg/day or drug level > 0.6 mEq/L for at least 4 weeks.

|  | **^HV (N=25)^** | **^TRD (N=39)^** | **^Overall (N=64)^** |
| --- | --- | --- | --- |
| **^Age^** | | | |
| ^Mean (SD)^ | ^34.0 (10.6)^ | ^36.3 (10.1)^ | ^35.4 (10.3)^ |
| **^Sex^** | | | |
| ^F^ | ^16 (64.0%)^ | ^23 (59.0%)^ | ^39 (60.9%)^ |
| ^M^ | ^9 (36.0%)^ | ^16 (41.0%)^ | ^25 (39.1%)^ |
| **^BMI^** | | | |
| ^Mean (SD)^ | ^27.4 (4.51)^ | ^27.5 (6.44)^ | ^27.5 (5.73)^ |
| **^Race or Ethnicity^** | | | |
| ^Asian^ | ^1 (4.0%)^ | ^1 (2.6%)^ | ^2 (3.1%)^ |
| ^Black^ | ^6 (24.0%)^ | ^2 (5.1%)^ | ^8 (12.5%)^ |
| ^Hispanic^ | ^1 (4.0%)^ | ^0 (0%)^ | ^1 (1.6%)^ |
| ^Multiple^ | ^1 (4.0%)^ | ^2 (5.1%)^ | ^3 (4.7%)^ |
| ^White^ | ^16 (64.0%)^ | ^33 (84.6%)^ | ^49 (76.6%)^ |
| **^Baseline MADRS^** | | | |
| ^Mean (SD)^ | ^1.48 (1.64)^ | ^33.4 (4.52)^ | ^20.9 (16.1)^ |
| **^TRD Treatment History (N=39)^** | | | |
| **^Total Failed Antidepressant Trials (Mean (SD))^** | | ^4.95 (2.93)^ | |
| **^Failed Augmentation Trials (Mean (SD))^** | | ^3.32 (3.03)^ | |
| **^Received Lithium Augmentation ( (n, (%))^** | | ^16 (41.0%)^ | |
| **^Prior ECT Treatment (n, (%))^** | | ^10 (25.6%)^ | |
| **^Past Suicide Attempts^** ^(^**^Mean (SD))^** | | ^0.474 (0.725)^ | |
| **^Past Hospital Admissions^** ^(^**^Mean (SD))^** | | ^1.45 (1.72)^ | |
| **^History of Trauma, Abuse, or Neglect^** | | ^18 (46.2%)^ | |
| **^History of Polytrauma^** | | ^6 (15.4%)^ | |
| **^Illness Duration (Mean Years (SD))^** | | ^22.1 (11.6)^ | |
| **^Currently Unemployed (n, (%))^** | | ^33 (84.6%)^ | |
| **^On Disability (n, (%))^** | | ^4 (10.3%)^ | |
| **^Family History of Mood Disorder (n, (%))^** | | ^32 (82.1%)^ | |
| **^Placebo Response Rates in Study (NCT00088699)^** | | | |
| ^Any >50% Improvement in MADRS (4hr to 3d) after Placebo^ | | ^0 (0%)^ | |
| ^Any >25% Improvement in MADRS (4hr to 3d) after Placebo^ | | ^1(2.8%)^ | |

Abbreviations: TRD: treatment-resistant depression; HV: healthy volunteer; BMI: body-mass index; MADRS: Montgomery-Åsberg Depression Rating Scale; ECT: electroconvulsive therapy.

**Supplementary Table S4. Demographic and Clinical Characteristics by Study Cohort and Dataset.** Demographic and clinical characteristics are presented separately for each cohort and diagnostic group contributing data to the analyses. Cohorts derived from the KMOA randomized crossover trial (NCT00088699; blood RNA, MEG, plasma cytokines) may overlap, as individual patients contributed data across multiple modalities; iPSC and CSF cohorts are independent. Age, sex, BMI, and race/ethnicity are reported for all cohorts; clinical and illness-severity variables are reported for TRD cohorts only (“—" indicates not applicable for HV cohorts). BMI was not collected (NC) for iPSC-HVs per protocol and is not reported (NR) in the publication for CSF-HV participants [8].

|  | iPSC-TRD (N=5) | iPSC-HV (N=5) | CSF-HV (N=9) | Blood RNA-TRD (N=16) | Blood RNA-HV (N=11) | MEG-TRD (N=30) | MEG-HV (N=25) | Cytokine-TRD (N=39) | Cytokine-HV (N=25) |
| --- | --- | --- | --- | --- | --- | --- | --- | --- | --- |
|  | | | | | | | | | |
| Age (years) Mean (SD) | 42 (9.5) | 37 (20) | 27 (6.4) | 39 (13) | 38 (13) | 36 (9.9) | 34 (11) | 36 (10) | 34 (11) |
| Sex |  |  |  |  |  |  |  |  |  |
| Male | 0 (0%) | 0 (0%) | 5 (56%) | 6 (38%) | 2 (18%) | 12 (40%) | 9 (36%) | 16 (41%) | 9 (36%) |
| Female | 5 (100%) | 5 (100%) | 4 (44%) | 10 (62%) | 9 (82%) | 18 (60%) | 16 (64%) | 23 (59%) | 16 (64%) |
| BMI Mean (SD) | 26 (3.6) | NC | NR | 29 (8.6) | 28 (3.5) | 27 (6.4) | 27 (4.5) | 27 (6.4) | 27 (4.5) |
| Race or Ethnicity |  | | | | | | | | |
| Black | 1 (20%) | 0 (0%) | 3 (33%) | 0 (0%) | 3 (27%) | 2 (7%) | 6 (24%) | 2 (5%) | 6 (24%) |
| White | 4 (80%) | 5 (100%) | 4 (44%) | 12 (75%) | 5 (45%) | 27 (90%) | 16 (64%) | 33 (85%) | 16 (64%) |
| Asian | 0 (0%) | 0 (0%) | 1 (11%) | 1 (6%) | 1 (9%) | 1 (3%) | 1 (4%) | 1 (3%) | 1 (4%) |
| Hispanic | 0 (0%) | 0 (0%) | 0 (0%) | 0 (0%) | 1 (9%) | 0 (0%) | 1 (4%) | 0 (0%) | 1 (4%) |
| Multiple | 0 (0%) | 0 (0%) | 1 (11%) | 2 (12%) | 1 (9%) | 0 (0%) | 1 (4%) | 2 (5%) | 1 (4%) |
| Baseline MADRS Mean (SD) | 33 (5.1) | — | — | 33 (4.2) | — | 33 (4.9) | — | 33 (4.5) | — |
| Total Failed AD Trials Mean (SD) | 7.2 (4.9) | — | — | 5.8 (3.0) | — | 4.6 (2.8) | — | 4.9 (2.9) | — |
| Failed Augmentation Trials Mean (SD) | 4.4 (3.4) | — | — | 4.1 (2.8) | — | 3.1 (3.0) | — | 3.3 (3.0) | — |
| Received Lithium Augmentation (n%) | 2 (40%) | — | — | 7 (44%) | — | 13 (43%) | — | 16 (41%) | — |
| Prior ECT Treatment (n %) | 1 (20%) | — | — | 6 (38%) | — | 7 (23%) | — | 10 (26%) | — |
| Past Suicide Attempts Mean (SD) | 0.40 (0.55) | — | — | 0.40 (0.63) | — | 0.50 (0.73) | — | 0.47 (0.73) | — |
| Past Hospital Admissions Mean (SD) | 1.4 (1.9) | — | — | 2.1 (1.9) | — | 1.5 (1.7) | — | 1.4 (1.7) | — |
| History of Trauma, Abuse, or Neglect( n %) | 2 (40%) | — | — | 7 (44%) | — | 13 (43%) | — | 18 (46%) | — |
| History of Polytrauma | 1 (20%) | — | — | 1 (6%) | — | 5 (17%) | — | 6 (15%) | — |
| Illness Duration (yrs) Mean (SD) | 28 (15) | — | — | 24 (14) | — | 22 (12) | — | 22 (12) | — |
| Currently Unemployed (n%) | 0 (0%) | — | — | 13 (81%) | — | 27 (90%) | — | 33 (85%) | — |
| On Disability (n%) | 0 (0%) |  |  | 3 (19%) |  | 2 (7%) |  | 4 (10%) |  |
| Family History of Mood Disorder (n%) | 4 (80%) | — | — | 11 (69%) | — | 26 (87%) | — | 32 (82%) | — |
| Any PRN Anti-Inflammatory Medication Use** (n%) | — | — | 0 (0%) | 15 (94%) |  | 21 (70%) |  | 28 (72%) |  |
| Comorbid Immune-Related Diagnosis (n%) | 0 (0%) | 0 (0%) | 0 (0%) | 3 (19%) | — | 3 (10%) | — | 4(10%) | — |

**The variable “any PRN anti-inflammatory medication use” reflects any administration of a non-scheduled anti-inflammatory agent within a −7/+3 day peri-infusion lookback window; sensitivity analyses examining the influence of medication exposure (including standardized dosage and frequency) and immune-relevant comorbidities on primary cytokine and antidepressant outcomes from **Figure 4** appear in **Figures S9 and S10**. Continuous variables are presented as mean (SD) and categorical variables as n (%). The field “Polytrauma” indicates multiple lifetime instances of psychological trauma, abuse, or neglect as indicated by the SCID-V interview. *Abbreviations:* BMI: body-mass index; MEG: magnetoencephalography; TRD: treatment-resistant depression; HV: healthy volunteer; MADRS: Montgomery-Åsberg Depression Rating Scale; AD: antidepressant; ECT: electroconvulsive therapy; iPSC; induced pluripotent stem cell; CSF: cerebrospinal fluid; KMOA: ketamine mechanism of action trial (NCT00088699).

**Supplementary Table S5: *BayesPrism* single-cell reference dataset cell types and corresponding marker genes.** This table provides the 48 immune cell populations used for deconvolution analysis from the Hao and colleagues reference dataset (n=161,764 peripheral blood mononuclear cells (PBMCs) from eight healthy donors) [21]. Cell types are listed with their expanded labels, Cell Ontology (OBO) IDs for standardized classification, and the top 10 marker genes defining each population. Marker genes were identified through differential expression analysis in the original single-cell dataset and validated using oligonucleotide antibody-derived tags. Bold formatting indicates the NK_3 population, which displayed canonical NKT markers and showed the most pronounced functional shifts in ketamine responders. Cell types with fewer than 30 cells in the reference were excluded from analysis per BayesPrism recommendations.

Adapted from: https://azimuth.hubmapconsortium.org/references/

| **Label** | **Expanded Label** | **OBO Ontology ID** | **Top Markers From Azimuth/Hao et al. [21]** |
| --- | --- | --- | --- |
| ASDC_mDC | AXL+ myeloid dendritic cell | myeloid dendritic cell | AXL, LILRA4, SCN9A, CLEC4C, LTK, PPP1R14A, LGMN, SCT, IL3RA, GAS6 |
| ASDC_pDC | AXL+ plasmacytoid dendritic cell | plasmacytoid dendritic cell | LILRA4, CLEC4C, SCT, EPHB1, AXL, PROC, LRRC26, SCN9A, LTK, DNASE1L3 |
| B intermediate kappa | Intermediate B-cell, kappa light chain | mature B-cell | MS4A1, IGKC, IGHM, LINC01857, MARCKS, IGHD, TNFRSF13B, CD24, FCRL2, BANK1 |
| B intermediate lambda | Intermediate B-cell, lambda light chain | mature B-cell | MS4A1, IGLC2, IGHM, CD79A, IGLC3, IGHD, BANK1, TNFRSF13C, CD22, TNFRSF13B |
| B memory kappa | Memory B-cell, kappa light chain | memory B-cell | BANK1, IGKC, LINC01781, MS4A1, SSPN, CD79A, RALGPS2, TNFRSF13C, LINC00926 |
| B memory lambda | Memory B-cell, lambda light chain | memory B-cell | BANK1, IGLC2, MS4A1, IGLC3, COCH, TNFRSF13C, IGHA2, BLK, TNFRSF13B, LINC01781 |
| B naive kappa | Naive B-cell, kappa light chain | naive B-cell | IGHM, TCL1A, IGHD, IGHG3, CD79A, IL4R, CD37, MS4A1, IGKC |
| B naive lambda | Naive B-cell, lambda light chain | naive B-cell | IGHM, IGLC2, IGHD, IGLC3, CD79A, CXCR4, MS4A1, IL4R, TCL1A, CD79B |
| CD14 Mono | CD14+ Monocyte | CD14-positive, CD16-negative classical monocyte | S100A9, CTSS, LYZ, CTSD, S100A8, VCAN, CD14, FCN1, S100A12, MS4A6A |
| CD16 Mono | CD16+ Monocyte | CD14-low, CD16-positive monocyte | LST1, YBX1, AIF1, FCGR3A, NAP1L1, MS4A7, FCER1G, TCF7L2, COTL1, CDKN1C |
| CD4 CTL | CD4+ Cytotoxic T | CD4-positive, alpha-beta cytotoxic T-cell | GZMH, CD4, GNLY, FGFBP2, IL7R, S100A4, GZMA, CST7, IL32, CCL5 |
| CD4 Naive | CD4+ Naive T | naive thymus-derived CD4-positive, alpha-beta T-cell | TCF7, CD4, NUCB2, LDHB, TRAT1, SARAF, FHIT, LEF1, CCR7, IL7R |
| CD4 Proliferating | CD4+ Proliferating T | activated CD4-positive, alpha-beta T-cell | MKI67, TYMS, PCLAF, TOP2A, CENPF, NUSAP1, CENPM, BIRC5, ZWINT, TPX2 |
| CD4 TCM_1 | CD4+ Central Memory T_1 | CD4-positive, alpha-beta memory T-cell | LTB, CD4, FYB1, IL7R, LIMS1, MAL, TMSB4X, TSHZ2, AP3M2, TRAC |
| CD4 TCM_2 | CD4+ Central Memory T_2 | CD4-positive, alpha-beta memory T-cell | CTLA4, MIR4435-2HG, TMSB4X, CD28, CDCA7, TMSB10, MAF, ITM2A, TRAC, CD27 |
| CD4 TCM_3 | CD4+ Central Memory T_3 | CD4-positive, alpha-beta memory T-cell | IL7R, ITGB1, LTB, S100A4, AQP3, TNFRSF4, IL32, TOB1, PDE4D, HOPX |
| CD4 TEM_1 | CD4+ Effector Memory T_1 | effector CD4-positive, alpha-beta T-cell | GZMK, IL7R, IL32, ITGB1, CCL5, GZMA, B2M, DUSP2, KLRB1, SYNE2 |
| CD4 TEM_2 | CD4+ Effector Memory T_2 | effector CD4-positive, alpha-beta T-cell | GZMK, CD4, TIGIT, IFNG-AS1, CD40LG, MALAT1, CD3G, CD3D, TRAC, CD3E |
| CD4 TEM_3 | CD4+ Effector Memory T_3 | effector CD4-positive, alpha-beta T-cell | IL7R, CCL5, NOSIP, KLRB1, SERINC5, AQP3, ITGA4, IL32, TRAC, LTB |
| CD4 TEM_4 | CD4+ Effector Memory T_4 | effector CD4-positive, alpha-beta T-cell | CCR9, KDF1, DPP4, CD244, SLC4A4, KLRB1, TMIGD2, CD40LG, IL7R, ODF2L |
| CD8 Naive | CD8+ Naive T | naive thymus-derived CD8-positive, alpha-beta T-cell | CD8B, LDHB, LEF1, LINC02446, CD8A, S100B, ID2, TCF7, VIM, CCR7 |
| CD8 Naive_2 | CD8+ Naive T_2 | naive thymus-derived CD8-positive, alpha-beta T-cell | CD8B, CCR5, CHI3L2, SOX4, CD8A, TNFRSF9, CD38, SIRPG, LRRN3, LEF1 |
| CD8 Proliferating | CD8+ Proliferating T | activated CD8-positive, alpha-beta T-cell | PCLAF, CD8B, TYMS, CD3D, CLSPN, CD3G, MKI67, TRAC, CHEK1, TK1 |
| CD8 TCM_1 | CD8+ Central Memory T_1 | CD8-positive, alpha-beta memory T-cell | CD8B, SELL, CD8A, LYAR, ITGB1, NELL2, DUSP2, IL7R, CCL5, LINC01871 |
| CD8 TCM_2 | CD8+ Central Memory T_2 | CD8-positive, alpha-beta memory T-cell | CD8B, C1orf162, IL7R, GATA3, YBX3, KRT1, CD8A, CTSW, INPP4B, LTB |
| CD8 TCM_3 | CD8+ Central Memory T_3 | CD8-positive, alpha-beta memory T-cell | CD8B, KLRB1, CD8A, HOPX, IL7R, KLRD1, CCL5, SCML4, LINC02446, TRAC |
| CD8 TEM_1 | CD8+ Effector Memory T_1 | effector CD8-positive, alpha-beta T-cell | GZMK, CD8B, CD8A, CCL5, NKG7, DUSP2, CST7, IL32 |
| CD8 TEM_2 | CD8+ Effector Memory T_2 | effector CD8-positive, alpha-beta T-cell | CD8A, CMC1, CD8B, CD160, GZMH, CST7, KLRD1, CCL5, TIGIT, KLRG1 |
| CD8 TEM_3 | CD8+ Effector Memory T_3 | effector CD8-positive, alpha-beta T-cell | GZMK, CD8A, ITGB1, CD8B, HOPX, CCL5, KLRD1, NKG7, GNLY, YBX3 |
| CD8 TEM_4 | CD8+ Effector Memory T_4 | effector CD8-positive, alpha-beta T-cell | GZMH, THEMIS, GNLY, CD8A, ITGB1, FGFBP2, CD2, GZMB, KLRD1, CD8B |
| CD8 TEM_5 | CD8+ Effector Memory T_5 | effector CD8-positive, alpha-beta T-cell | GZMH, GNLY, ZNF683, TRAC, KLRC2, TYROBP, CD8B, CD8A, TRGC2, GZMB |
| CD8 TEM_6 | CD8+ Effector Memory T_6 | effector CD8-positive, alpha-beta T-cell | TRGC2, CD8A, IFNG-AS1, CD8B, ZNF683, KLRC2, NCR3, IKZF2, DUSP2, RTKN2 |
| cDC1 | Conventional Dendritic Cell 1 | CD141-positive myeloid dendritic cell | WDFY4, C1orf54, CLEC9A, BATF3, CLNK, TSPAN33, FLT3, CADM1, IDO1, DNASE1L3 |
| cDC2_1 | Conventional Dendritic Cell 2_1 | CD1c-positive myeloid dendritic cell | FCER1A, CD14, CLEC10A, CTSS, ENHO, CD1C, MRC1, FCGR2B, PID1, IL13RA1 |
| cDC2_2 | Conventional Dendritic Cell 2_2 | CD1c-positive myeloid dendritic cell | FCER1A, BASP1, CD1C, CD74, CLEC10A, HLA-DPA1, ENHO, HLA-DPB1, PLD4, HLA-DQA1 |
| dnT_1 | Double-negative T_1 | CD4-negative, CD8-negative, alpha-beta intraepithelial T-cell | GZMK, NUCB2, CD8B, GPR183, TCF7, LYAR, MALAT1, C12orf57, LEF1, LDHB |
| dnT_2 | Double-negative T_2 | CD4-negative, CD8-negative, alpha-beta intraepithelial T-cell | AC004585.1, GPR183, FXYD2, NUCB2, CAV1, CD27, MYB, TMSB4X, GZMK, FGFR1 |
| Eryth | Erythroid cell | erythroid lineage cell | HBM, ALAS2, HBD, AHSP, SLC4A1, TRIM58, SELENBP1, CA1, IFIT1B, SNCA |
| gdT_1 | gamma-delta T_1 | gamma-delta T-cell | TRDC, TRGC1, TRGV9, TRDV2, KLRD1, IL7R, KLRC1, DUSP2, GNLY, KLRG1 |
| gdT_2 | gamma-delta T_2 | gamma-delta T-cell | KLRC2, CD3G, KIR3DL2, CD3D, TRDC, TRDV1, ZNF683, KLRC1, TRGC1, GZMH |
| gdT_3 | gamma-delta T_3 | gamma-delta T-cell | RTKN2, TRDC, TRGC2, LEF1, IKZF2, SOX4, ZNF331, ARID5B, NUCB2, CRTAM |
| gdT_4 | gamma-delta T_4 | gamma-delta T-cell | TRDC, TIGIT, KLRC2, TRGC2, IKZF2, GCSAM, FCRL6, TRDV1, CST7, CMC1 |
| HSPC | Hematopoietic stem and progenitor cell | hematopoietic precursor cell | CDK6, SOX4, PRSS57, AC084033.3, ANKRD28, FAM30A, MYB, EGFL7, SPINK2, SMIM24 |
| ILC | Innate Lymphoid Cell | innate lymphoid cell | KIT, TRDC, IL1R1, SOX4, TNFRSF18, TYROBP, TNFRSF4, FCER1G, IL2RA, GATA3 |
| MAIT | Mucosal associated invariant T | mucosal invariant T-cell | KLRB1, NKG7, GZMK, SLC4A10, NCR3, CTSW, IL7R, KLRG1, CEBPD, DUSP2 |
| NK Proliferating | Proliferating Natural Killer | natural killer cell | STMN1, KLRF1, TYMS, FCER1G, PCNA, TYROBP, CLSPN, TRDC, PCLAF, SMC2 |
| NK_1 | CD56-dim Natural Killer_1 | CD16-positive, CD56-dim natural killer cell, human | FGFBP2, KLRC2, GNLY, S100A4, CD3E, CST7, LGALS1, PRF1, NKG7, GZMB |
| NK_2 | CD56-dim Natural Killer_2 | CD16-positive, CD56-dim natural killer cell, human | NKG7, FCER1G, PRF1, KLRB1, SPON2, GZMB, FGFBP2, IGFBP7, CST7, B2M |
| **NK_3** | **CD56-dim Natural Killer_3** | **CD16-positive, CD56-dim natural killer cell, human** | **KLRF1, CCL5, TRDC, SYNE2, KLRC1, CMC1, XCL2, KLRB1, KLRD1, IL2RB** |
| NK_4 | CD56-dim Natural Killer_4 | CD16-positive, CD56-dim natural killer cell, human | XCL2, SELL, XCL1, GZMK, KLRC1, SPTSSB, KLRF1, IL2RB, TCF7, TRDC |
| NK_CD56bright | CD56-bright Natural Killer | CD16-negative, CD56-bright natural killer cell, human | XCL2, GPR183, SELL, IL2RB, CD44, GZMK, KLRF1, TPT1, KLRC1, XCL1 |
| pDC | Plasmacytoid Dendritic Cell | plasmacytoid dendritic cell | CCDC50, UGCG, TCF4, LILRA4, IRF8, IL3RA, PLD4, IRF7, SERPINF1, ITM2C |
| Plasma | Plasma cell | plasma cell | MZB1, JCHAIN, TNFRSF17, ITM2C, DERL3, TXNDC5, POU2AF1, IGHA1, TXNDC11, CD79A |
| Plasmablast | Plasmablast | plasmablast | TYMS, TNFRSF17, SHCBP1, TK1, KNL1, ASPM, TXNDC5, TPX2, RRM2, BIRC5 |
| Platelet | Platelet | platelet | GNG11, PPBP, NRGN, PF4, CAVIN2, TUBB1, HIST1H2AC, PRKAR2B, CLU, F13A1 |
| Treg Memory | Memory Regulatory T | regulatory T-cell | RTKN2, B2M, TIGIT, FCRL3, S100A4, AC133644.2, CTLA4, FOXP3, IKZF2, TRAC |
| Treg Naive | Naive Regulatory T | regulatory T-cell | RTKN2, LEF1, FOXP3, C12orf57, IL2RA, TOMM7, CCR7, TRAC, CD4, LDHB |

**Supplementary Figures**

**Supplementary Figure S1: Correlation of Gene Expression Across Rapid-Acting Antidepressants.** Pairwise correlation matrices demonstrating the convergent transcriptional responses across pharmacologically distinct rapid-acting antidepressants in induced pluripotent stem cell (iPSC)-derived neurons for all genes (left; n = 22,810), differentially expressed genes (DEGs) across all samples (middle; n = 3157), and DEGs common to all samples (right; n = 26). Pearson correlation coefficients were calculated versus vehicle control. All pairwise correlations showed high concordance (r = 0.70-0.88; all p_FDR < 2e-16) for DEGs, suggesting shared downstream molecular pathways despite distinct primary receptor targets. The strongest correlations were observed between the psychedelic compounds LSD and psilocybin (r = 0.88), while ketamine showed the lowest but still substantial correlation with other compounds (r = 0.70-0.75), potentially due to fewer samples sequenced. Color intensity represents correlation strength, with warmer colors indicating stronger positive correlations.

**Supplementary** **Figure S2. Protein-protein interaction (PPI) network of the 103-protein rapid-acting antidepressant (RAAD) signatures.** STRING network diagram of 103 proteins identified across induced pluripotent stem cell (iPSC) and cerebrospinal fluid (CSF) datasets in at least one timepoint for three or more rapid-acting antidepressants (ketamine, psilocybin, LSD, (2*R*,6*R*-hydroxynorketamine (HNK)). Edges represent high confidence predicted or experimentally validated interactions, and nodes are color-coded by functional or interaction clusters. Central hubs in the network include immune modulators, kinase signaling molecules, and neurodevelopmental factors, highlighting a coordinated protein network potentially underlying RAAD response.

**Supplementary Figure S3. Replication of *SPECC1L-ADORA2A* read-through transcript in ketamine responders.** (**a**) Integrative Genomics Viewer (IGV) visualization confirming read-through transcription from *SPECC1L* into *ADORA2A* in a subset of representative samples shown to highly express *SPECC1L-ADORA2A*, including in individuals with treatment-resistant depression (TRD) and healthy volunteers (HVs) both pre- and post-ketamine. (**b-c**) Volcano plots showing differential gene expression between responders and non-responders at baseline **(b)** and pre-post treatment **(c)** using Salmon quantification. *SPECC1L-ADORA2A* and *ADORA2A* are highlighted, with the fusion transcript showing nominal significance (p=0.07), consistent with the primary analysis. (**d**) Mean effective transcript lengths demonstrating that *SPECC1L-ADORA2A* (7085 bp) was approximately three-fold longer than canonical *ADORA2A* (2185 bp), characteristic of a read-through fusion event. logFC, log2 fold change.

**Supplementary Figure S4. Replication of *SPECC1L-ADORA2A* read-through transcript in post-mortem subgenual anterior cingulate cortex (sgACC).** (**a**) Integrative Genomics Viewer (IGV) visualization confirming read-through transcription from *SPECC1L* into *ADORA2A* in a subset of representative samples shown to highly express *SPECC1L-ADORA2A*, including both individuals with major depressive disorder (MDD) and healthy volunteers (HVs) from publicly available sequencing data (dbGAP: phs000979.v2.p2). (**b-c**) Box plots showing differential gene expression (TPM) between MDD patients and HVs for *SPECC1L-ADORA2A* **(b)** and *ADORA2A* **(c)** using Stringtie quantification. (**d**) Mean effective transcript lengths demonstrating that *SPECC1L-ADORA2A* (6204 bp) is approximately three-fold longer than canonical *ADORA2A* (1189 bp), characteristic of a read-through fusion event.

**Supplementary Figure****S5. Volcano plots comparing natural killer T-cell (NKT) outlier patients (n=2) versus remaining patients across deconvolution and complete blood count (CBC) metrics**. Left panel shows deconvolution-derived cell type proportions; right panel shows CBC parameters. The two patients identified as NKT outliers demonstrated values within the normal range for most measured parameters, with notable differences primarily in platelet count and select immune cell populations (B-cells, common lymphoid progenitors, and CD4+ memory T-cells only). The limited scope of differences suggests that excluding these patients' deconvolution data based solely on elevated NKT cell proportions would not be justified, as their overall hematologic and immunologic profiles remain largely consistent with the broader patient cohort.

**Supplementary Figure S6. Predicted changes in cell-type specific functional activity from single-cell deconvolution of bulk transcriptomes using *BayesPrism* and GO Molecular Function enrichment.** *BayesPrism* deconvolution was performed using a single-cell reference dataset obtained from Hao and colleagues [21], which included 161,764 immune cells across 57 subpopulations from eight healthy donors. After filtering for subtypes with sufficient representation, 48 cell types were used. Gene set enrichment analysis was performed using zenith with GO Molecular Function terms, applied to cell-type-specific model coefficients. Cell types were collapsed to include only those with at least one significantly enriched gene set (false discovery rate (FDR) < 0.05). **(a)** At baseline, B-cell subsets were broadly enriched for upregulated functions, while natural killer cell (NK) subsets—especially NK_3, a canonical natural killer T-cell (NKT)-like population—exhibited widespread downregulation of molecular functions. **(b)** Post-ketamine, these patterns reversed in responders: NK_3 activity increased, with enrichment for protein serine/threonine kinase activity, histone modification, and nuclear receptor binding, suggestive of mechanistic target of rapamycin (mTOR) signaling and epigenetic activation. Conversely, OXPHOS-related pathways were downregulated, suggesting a glycolytic metabolic shift. Regulatory T-cells (Treg memory) also showed increased activity in responders. See **Table S5** for marker genes and a full description of cell types and enrichment results.

**Supplementary Figure S7. Plasma interleukin-15 (IL-15) concentrations in detectable samples following ketamine administration. (A)** Bio-RAD limit of detection (LOD) data for individual analytes in the multiplex assay, showing that IL-15 had the highest LOD out of all analytes by a wide margin, which likely contributed to low detection rate at the 4:1 dilution used to accommodate other analytes. (**B**) Longitudinal plasma IL-15 levels measured at baseline, 4 hours, Day 1, and Day 3 post-infusion in participants with treatment-resistant depression (TRD) and healthy volunteers (HVs; data drawn from NCT00088699). Due to the cytokine's rapid clearance (<1 hour half-life), high membrane bound ratio, and tight post-transcriptional regulation, approximately 80% of samples were below the limit of detection. Among detectable samples (representing various timepoints across 18 different patients), IL-15 levels showed numerical increases following ketamine administration in both groups, consistent with transcriptomic upregulation observed in induced pluripotent stem cell (iPSC) neurons and cerebrospinal fluid (CSF) proteomics. LOD/quantification and experimental standard data can be found in the Supplementary data file (FIGURE_4_CYTOKINES.xlsx).

**Supplementary Figure S8. Magnetoencephalography (MEG) gamma power correlates of plasma monocyte chemoattractant protein-1 (MCP-1) concentrations.** Brain surface maps displaying the relationship between plasma MCP-1/CCL2 levels and resting-state MEG gamma power (30-50 Hz) across all participants with both cytokine data and MEG (n=31). Linear mixed-effects models revealed that higher MCP-1 concentrations were associated with significantly reduced gamma power in bilateral frontal cortical regions and the subgenual anterior cingulate cortex (main effect p_uncorrected < 0.05). No regions survived cluster-wise false discovery rate (FDR) correction.

**Supplementary Figure S9. NSAID Exposure and Baseline Cytokine Levels by NSAID Use and Immune Comorbidity Status.** (A) Mean daily Assessment of SpondyloArthritis International Society NSAID (ASAS-NSAID) equivalence score per patient per infusion, calculated over a −7/+3 day peri-infusion window. Annotations indicate counts of NSAID users and non-users within each diagnostic group (treatment-resistant depression (TRD), healthy volunteer (HV)) per infusion. (B) Baseline log-transformed cytokine levels at Infusion 1 comparing NSAID users and non-users (Wilcoxon rank-sum; uncorrected p in orange, Benjamini-Hochberg-corrected p below; p<0.05 threshold). Individual data points are colored by immune comorbidity diagnosis; grey points indicate no comorbidity.

**Supplementary Figure S10. Sensitivity Analyses: Effects of NSAID Exposure and Immune Comorbidity on Cytokine Levels and Cytokine Moderation of Antidepressant Response.** **(A)** Standardized effect sizes for Assessment of SpondyloArthritis International Society NSAID (ASAS-NSAID) score on baseline cytokine levels (Baseline) and Treatment × ASAS interaction on cytokine trajectories (Full Study), controlling for age, sex, and body-mass index (BMI). **(B)** Equivalent models substituting immune comorbidity flag (exploratory; n=4). **(C–D)** Cytokine × Treatment interaction effects on Montgomery-Asberg Depression Rating Scale (MADRS), with ASAS score **(C)** or comorbidity **(D)** entered as covariates; left panels show baseline cytokine interactions, right panels show full-study interactions. **(E–F**) Fixed effects of ASAS score **(E)** and comorbidity **(F)** on MADRS score extracted from the same models. **(G–I)** Combined models including both ASAS score and comorbidity simultaneously: cytokine × treatment interactions **(G)**, ASAS fixed effects **(H)**, and comorbidity fixed effects **(I)**. All models controlled for age, sex, BMI, infusion order, and timepoint. p<0.05 labeled in respective panel colors; comorbidity analyses are exploratory, given that only n=4 patients had comorbid immune-related illnesses.

**Supplementary Discussion**

*Extended Clinical Correlates Supporting the IL-15/IL-7 Framework*

Our iPSC-CSF overlap network showed significant enrichment of bone-related terms alongside IL-15 production (**Fig.2e**). Clinical depression has consistently been linked with reduced bone mineral density [45, 46], and a previous study from our laboratory found that ketamine exerted putatively beneficial shifts in bone metabolic markers in individuals with TRD [47]. This may relate directly to IL-15 signaling, as transgenic mice overexpressing circulating IL-15 also display increased bone mineral density [48]. In addition, insulin signaling was downregulated at baseline in the blood transcriptomics of ketamine responders, elevated in our integrated iPSC-CSF network, and previously shown by our laboratory to be increased nearly four-fold in CSF for at least 12 hours post-ketamine [8]. Although IL-15 crosses the blood-brain barrier in limited quantities, it primarily activates hypothalamic c-Fos *in vivo* [49]—a master regulator of insulin response [50]—and stimulates the release of insulin-sensitizing adiponectin [51]. In a larger TRD cohort (n=80), another study from our laboratory found that lower baseline adiponectin levels significantly predicted antidepressant response to ketamine [52], mirroring the IL-15 deficiency/reversal proposed here. Lastly, IL-15 stimulates MCP-1 release [53], which was observed in our iPSC-CSF network in addition to being the strongest predictor of antidepressant *non-response* in peripheral blood. That IL-4/IFN-γ ratios—which govern whether MCP-1 is released downstream of IL-15 [53]—emerged as the strongest predictor of clinical response further supports the importance of IL-15 pathways.

*Comparison with Prior Ketamine Transcriptomic Approaches*

The ketamine blood transcriptomics approach here differs from prior work [58]. This study assessed early RNA changes (0–4 hours), hypothesizing that they likely precede peak clinical response (24 hours post-infusion [59]). Hemoglobin depletion was also used, whereas 38% of raw reads in a study by Cathomas and colleagues [58] were derived from hemoglobin genes. Each approach has tradeoffs: non-depletion risks reduced sensitivity for disease-relevant genes [60], while depletion can mask metabolic pathway expression [61]. Cathomas and colleagues [58] found that metabolic (glutamate) and cyclic adenosine monophosphate (cAMP) signaling differentiated responders; both pathways are clearly established ketamine mechanisms [47, 58, 62]. Rarer immune transcripts were found here, likely due to increased read availability alongside metabolic masking [61]. Importantly, both approaches identified bone-related signals associated with response, underscoring convergent biology despite methodological differences.

**R Package Versions**

| **Package** | **Version** |
| --- | --- |
| dplyr | 1.2.0 |
| tidyverse | 2.0.0 |
| readxl | 1.4.5 |
| openxlsx | 4.2.8.1 |
| purrr | 1.2.1 |
| ggplot2 | 4.0.2 |
| ggrepel | 0.9.7 |
| gridExtra | 2.3 |
| grid | 4.5.0 |
| cowplot | 1.2.0 |
| patchwork | 1.3.2 |
| tidytext | 0.4.3 |
| ggVennDiagram | 1.5.7 |
| viridis | 0.6.5 |
| viridisLite | 0.4.3 |
| ggbeeswarm | 0.7.3 |
| lme4 | 1.1.38 |
| lmerTest | 3.2.1 |
| effectsize | 1.0.1 |
| ggeffects | 2.3.2 |
| emmeans | 2.0.2 |
| edgeR | 4.6.3 |
| limma | 3.66.0 |
| variancePartition | 1.38.1 |
| biomaRt | 2.64.0 |
| EnhancedVolcano | 1.26.0 |
| clusterProfiler | 4.16.0 |
| org.Hs.eg.db | 3.21.0 |
| ComplexHeatmap | 2.24.1 |
| zenith | 1.10.0 |
| pheatmap | 1.0.13 |
| RColorBrewer | 1.1.3 |
| knitr | 1.51 |
| kableExtra | 1.4.0 |
| igraph | 2.2.2 |
| STRINGdb | 2.20.0 |
| ggraph | 2.2.2 |
| tidygraph | 1.3.1 |

**Supplementary References**

1.	Bardy C, van den Hurk M, Eames T, Marchand C, Hernandex RV, Kellogg M *et al.* Neuronal medium that supports basic synaptic functions and activity of human neurons in vitro. *Proc Natl Acad Sci* 2015; **112:** E2725-E2734.

2.	Cavalleri L, Merlo Pich E, Millan MJ, Chiamulera C, Kunath T, Spano PF *et al.* Ketamine enhances structural plasticity in mouse mesencephalic and human iPSC-derived dopaminergic neurons via AMPAR-driven BDNF and mTOR signaling. *Mol Psychiatry* 2017; **23**(4)**:** 812-823.

3.	Ly C, Greb AC, Cameron LP, Wong JM, Barragan EV, Wilson PC *et al.* Psychedelics promote structural and functional neural plasticity. *Cell Rep* 2018; **23**(11)**:** 3170-3182.

4.	Dobin A, Davis CA, Schlesinger F, Drenkow J, Zaleski C, Jha S *et al.* STAR: ultrafast universal RNA-seq aligner. *Bioinformatics* 2013; **29:** 15-21.

5.	Liao Y, Smyth GK, Shi W. featureCounts: an efficient general purpose program for assigning sequence reads to genomic features. *Bioinformatics* 2014; **30:** 923-930.

6.	Hoffman GE, Roussos P. Dream: powerful differential expression analysis for repeated measures designs. *Bioinformatics* 2021; **37**(2)**:** 192-201.

7.	Hoffman GE, Schadt EE. variancePartition: interpreting drivers of variation in complex gene expression studies. *BMC Bioinformatics* 2016; **17:** 483.

8.	Moaddel R, Farmer CA, Yavi M, Kadriu B, Zhu M, Fan J *et al.* Cerebrospinal fluid exploratory proteomics and ketamine metabolite pharmacokinetics in human volunteers after ketamine infusion. *iScience* 2023; **26**(12)**:** 108527.

9.	Yeager MP, Lunt P, Arruda J, Whalen K, Rose R, DeLeo JA. Cerebrospinal fluid cytokine levels after surgery with spinal or general anesthesia. *Reg Anesth Pain Med* 1999; **24:** 557-562.

10.	Sackeim HA. The definition and meaning of treatment-resistant depression. *J Clin Psychiatry* 2001; **62 (Suppl 16):** 10-17.

11.	Montgomery SA, Åsberg M. A new depression scale designed to be sensitive to change. *Br J Psychiatry* 1979; **134**(4)**:** 382-389.

12.	Bioinformatics B. Fast QC: A quality control tool for high throughput sequence data. Available at: <https://www.bioinformatics.babraham.ac.uk/projects/fastqc/>.

13.	Chen S, Zhou Y, Chen Y, Gu J. fastp: an ultra-fast all-in-one FASTQ preprocessor. *Bioinformatics* 2018; **34:** i884-i890.

14.	Kim D, Paggi JM, Park C, Bennett C, Salzberg SL. Graph-based genome alignment and genotyping with HISAT2 and HISAT-genotype. *Nat Biotechnol* 2019; **37**(8)**:** 907-915.

15.	Pertea M, Kim D, Pertea GM, Leek JT, Salzber SL. Transcript-level expression analysis of RNA-seq experiments with HISAT, StringTie and Ballgown. *Nat Protoc* 2016; **11:** 1650-1667.

16.	Okonechnikov K, Conesa A, García-Alcalde F. Qualimap 2: advanced multi-sample quality control for high-throughput sequencing data. *Bioinformatics* 2016; **32:** 292-294.

17.	Krämer A, Green J, Pollard J, Jr., Tugendreich S. Causal analysis approaches in Ingenuity Pathway Analysis. *Bioinformatics* 2014; **30**(4)**:** 523-530.

18.	Aran D, Hu Z, Butte AJ. xCell: digitally portraying the tissue cellular heterogeneity landscape. *Genome Biol* 2017; **18**(1)**:** 220.

19.	Sturm G, Finotello F, Petitprez F, Zhang JD, Baumbach J, Fridman WH *et al.* Comprehensive evaluation of transcriptome-based cell-type quantification methods for immuno-oncology. *Bioinformatics* 2019; **35**(14)**:** i436-i445.

20.	Chu T, Wang Z, Pe’er D, Danko CG. Cell type and gene expression deconvolution with BayesPrism enables Bayesian integrative analysis across bulk and single-cell RNA sequencing in oncology. *Nat Cancer* 2022; **3**(4)**:** 505-517.

21.	Hao Y, Hao S, Andersen-Nissen E, Mauck WM, Zheng S, Butler A *et al.* Integrated analysis of multimodal single-cell data. *Cell* 2021; **184**(13)**:** 3573-3587.e3529.

22.	Kenward MG, Roger JH. The use of baseline covariates in crossover studies. *Biostatistics* 2010; **11**(1)**:** 1-17.

23.	Ben-Shachar MS, Lüdecke D, Makowski D. effectsize: Estimation of effect size indices and standardized parameters. *J Open Source Softw* 2020; **5**(56)**:** 2815.

24.	Álvarez-Soria MA, Largo R, Santillana J, Sánchez-Pernaute O, Calvo E, Hernández M *et al.* Long term NSAID treatment inhibits COX‐2 synthesis in the knee synovial membrane of patients with osteoarthritis: differential proinflammatory cytokine profile between celecoxib and aceclofenac. *Ann Rheum Dis* 2006; **65:** 998-1005.

25.	Mannava S, Whitney KE, Kennedy MI, King J, Dornan GJ, Klett K *et al.* The influence of naproxen on biological factors in leukocyte-rich platelet-rich plasma: A prospective comparative study. *Arthroscopy* 2019; **35:** 201-210.

26.	Simon LS, Weaver AL, Graham DY, Kivitz AJ, Lipsky PE, Hubbard RC *et al.* Anti-inflammatory and upper gastrointestinal effects of celecoxib in rheumatoid arthritis: a randomized controlled trial. *JAMA* 1999; **282:** 1921-1928.

27.	Türck D, Roth W, Busch U. A review of the clinical pharmacokinetics of meloxicam. *Br J Rheumatol* 1996; **35 (Suppl 1):** 13-16.

28.	Dougados M, Simon P, Braun J, Burgos-Vargas R, Maksymowych WP, Sieper J *et al.* ASAS recommendations for collecting, analysing and reporting NSAID intake in clinical trials/epidemiological studies in axial spondyloarthritis. *Ann Rheum Dis* 2011; **70:** 249-251.

29.	Vrba J, Robinson SE. Signal processing in magnetoencephalography. *Methods* 2021; **25:** 249-271.

30.	Schoergenhofer C, Schwameis M, Firbas C, Bartko J, Derhaschnig U, Mader RM *et al.* Single, very low rituximab doses in healthy volunteers - a pilot and a randomized trial: implications for dosing and biosimilarity testing. *Sci Rep* 2018; **8:** 124.

31.	Szałach ŁP, Ciesielska-Figlon K, Daca A, Cubała WJ, Lisowska KA. The effect of ketamine on the immune system in patients with treatment-resistant depression. *Int J Mol Sci* 2025; **26:** 7500.

32.	Aqrawi LA, Ivanchenko M, Björk A, Sepúlveda JIR, Imgenberg-Kreuz J, Kvarnström M *et al.* Diminished CXCR5 expression in peripheral blood of patients with Sjögren's syndrome may relate to both genotype and salivary gland homing. *Clin Exp Immunol* 2018; **192:** 259-270.

33.	Yue C, Wang N, Zhai H, Yuan Z, Cui Y, Quan J *et al.* Adenosine signalling drives antidepressant actions of ketamine and ECT. *Nature* 2026; **649:** 423-431.

34.	Akula N, Marenco S, Johnson K, Feng N, Zhu K, Schulmann A *et al.* Deep transcriptome sequencing of subgenual anterior cingulate cortex reveals cross-diagnostic and diagnosis-specific RNA expression changes in major psychiatric disorders. *Neuropsychopharmacology* 2021; **46:** 1364-1372.

35.	Simma N, Bose T, Kahlfuß S, Mankiewicz J, Lowinus T, Lühder F *et al.* NMDA-receptor antagonists block B-cell function but foster IL-10 production in BCR/CD40-activated B cells. *Cell Commun Signal* 2014; **12**(1)**:** 75.

36.	Ashkar AA, Rosenthal KL. Interleukin-15 and natural killer and NKT cells play a critical role in innate protection against genital herpes simplex virus type 2 infection. *J Virol* 2003; **77**(18)**:** 10168-10171.

37.	Gordy LE, Bezbradica JS, Flyak AI, Spencer CT, Dunkle A, Sun J *et al.* IL-15 regulates homeostasis and terminal maturation of NKT cells. *J Immunol* 2011; **187**(12)**:** 6335-6345.

38.	Apert C, Galindo-Albarrán AO, Castan S, Detraves C, Michaud H, McJannett N *et al.* IL-2 and IL-15 drive intrathymic development of distinct periphery-seeding CD4+Foxp3+ regulatory T lymphocytes. *Front Immunol* 2022; **13:** 965303.

39.	Gene set analysis following differential expression using linear (mixed) modeling with dream.

40.	Subramanian A, Tamayo P, Mootha VK, Mukherjee S, Ebert BL, Gillette MA *et al.* Gene set enrichment analysis: A knowledge-based approach for interpreting genome-wide expression profiles. *Proc Natl Acad Sci* 2005; **102**(43)**:** 15545-15550.

41.	Aaron RV, Ravyts SG, Carnahan ND, Bhattiprolu K, Harte N, McCaulley CC *et al.* Prevalence of depression and anxiety among adults with chronic pain: A systematic review and meta-analysis. *JAMA Netw Open* 2025; **8:** e250268.

42.	Bair MJ, Robinson RL, Katon W, Kroenke K. Depression and pain comorbidity: A literature review. *Arch Intern Med* 2003; **163:** 2433-2445.

43.	Bessler H, Cohen-Terica D, Djaldetti M, Sirota P. The effect of ibuprofen on cytokine production by mononuclear cells from schizophrenic patients. *Folia Biol (Praha)* 2017; **63:** 13-19.

44.	Bessler H, Ziyada S, Bergman M, Punsky I, Sirota L. Indomethacin and ibuprofen effect on IL-1ra production by mononuclear cells of preterm newborns and adults. *Biol Neonate* 2002; **82:** 73-77.

45.	Cizza G, Primma S, Csako G. Depression as a risk factor for osteoporosis. *Trends Endocrinol Metab* 2009; **20**(8)**:** 367-373.

46.	Wu Q, Magnus JH, Liu J, Bencaz AF, Hentz JG. Depression and low bone mineral density: a meta-analysis of epidemiologic studies. *Osteoporos Int* 2009; **20**(8)**:** 1309-1320.

47.	Kadriu B, Gold PW, Luckenbaugh DA, Lener MS, Ballard ED, Niciu MJ *et al.* Acute ketamine administration corrects abnormal inflammatory bone markers in major depressive disorder. *Mol Psychiatry* 2018; **23**(7)**:** 1626-1631.

48.	Quinn LS, Anderson BG, Strait-Bodey L, Stroud AM, Argilés JM. Oversecretion of interleukin-15 from skeletal muscle reduces adiposity. *Am J Physiol Endocrinol Metab* 2009; **296**(1)**:** E191-E202.

49.	He Y, Wu X, Khan RS, Kastin AJ, Cornelissen-Guillaume GG, Hsuchou H *et al.* IL-15 receptor deletion results in circadian changes of locomotor and metabolic activity. *Journal of molecular neuroscience: MN* 2010; **41**(2)**:** 315-321.

50.	Chan O, Sherwin RS. Hypothalamic regulation of glucose-stimulated insulin secretion. *Diabetes* 2012; **61**(3)**:** 564-565.

51.	Quinn LS, Strait-Bodey L, Anderson BG, Argilés JM, Havel PJ. Interleukin-15 stimulates adiponectin secretion by 3T3-L1 adipocytes: evidence for a skeletal muscle-to-fat signaling pathway. *Cell Biol Int* 2005; **29**(6)**:** 449-457.

52.	Machado-Vieira R, Gold PW, Luckenbaugh DA, Ballard ED, Richards EM, Henter ID *et al.* The role of adipokines in the rapid antidepressant effects of ketamine. *Mol Psychiatry* 2017; **22**(1)**:** 127-133.

53.	Badolato R, Ponzi AN, Millesimo M, Notarangelo LD, Musso T. Interleukin-15 (IL-15) induces IL-8 and monocyte chemotactic protein 1 production in human monocytes. *Blood* 1997; **90**(7)**:** 2804-2809.

54.	Kim K, Doi A, Wen B, Ng K, Zhao R, Cahan P *et al.* Epigenetic memory in induced pluripotent stem cells. *Nature* 2010; **467:** 285-290.

55.	Kyttälä A, Moraghebi R, Valensisi C, Kettunen J, Andrus C, Pasumarthy KK *et al.* Genetic variability overrides the impact of parental cell type and determines iPSC differentiation potential. *Stem Cell Reports* 2016; **6**(2)**:** 200-212.

56.	Thomas SM, Kagan C, Pavlovic B, Burnett J, Patterson K, Pritchard JK *et al.* Reprogramming LCLs to iPSCs results in recovery of donor-specific gene expression signature. *PLoS Genet* 2015; **11**(5)**:** e1005216.

57.	Scesa G, Adami R, Bottai D. iPSC preparation and epigenetic memory: Does the tissue origin matter? *Cells* 2021; **10:** 1470.

58.	Cathomas F, Bevilacqua L, Ramakrishnan A, Kronman H, Costi S, Schneider M *et al.* Whole blood transcriptional signatures associated with rapid antidepressant response to ketamine in patients with treatment resistant depression. *Transl Psychiatry* 2022; **12:** 12.

59.	Coyle CM, Laws KR. The use of ketamine as an antidepressant: a systematic review and meta-analysis. *Hum Psychopharmacol* 2015; **30**(3)**:** 152-163.

60.	Sheerin D, Lakay F, Esmail H, Kinnear C, Sansom B, Glanzmann B *et al.* Identification and control for the effects of bioinformatic globin depletion on human RNA-seq differential expression analysis. *Sci Rep* 2023; **13**(1)**:** 1859.

61.	Shin H, Shannon CP, Fishbane N, Ruan J, Zhou M, Balshaw R *et al.* Variation in RNA-Seq transcriptome profiles of peripheral whole blood from healthy individuals with and without globin depletion. *PLoS One* 2014; **9**(3)**:** e91041.

62.	Johnston JN, Kadriu B, Allen J, Gilbert JR, Henter ID, Zarate CAJ. Ketamine and serotonergic psychedelics: An update on the mechanisms and biosignatures underlying rapid-acting antidepressant treatment. *Neuropharmacology* 2023; **226:** 109422.
