# Supplementary material for: Convergent neuroimmune signaling underlying rapid antidepressant response to ketamine and psychedelics
Source: Mol Psychiatry. 2026 Jul 28;31(12):7251–63. doi: 10.1038/s41380-026-03777-z (PMC13736677; doi:10.1038/s41380-026-03777-z)
Supplement: Supplementary file 2 [file 41380_2026_3777_MOESM2_ESM.zip › DataUpload/Datafile_Description.docx]

**Files Included**

- **AnalysisPipeline.Rmd** — The complete annotated R Markdown analysis pipeline. Loads all data files, fits statistical models, and generates all figures. Be sure to change the file path.
- **FIG2IPSCData.xlsx** — Raw and processed data for Figure 2 (iPSC transcriptomics & protein network). Tabs include:
  - **HNK124, Psil1024, LSD24, KET24** — Differential expression top tables (Dream/limma) for each drug condition at 24h
  - **iPSCMetadata** — Sample/condition metadata for bulk RNA-seq
  - **CSFDEPsMoaddel2023** — Differential CSF protein expression from Moaddel et al. (2023)
  - **iPSCCSF103STRING** — The 103-protein iPSC–CSF overlap network gene list used for STRING/Cytoscape analysis
  - **CYTOSCAPE103** — Cytoscape network metrics (e.g., betweenness centrality, degree) for the 103-protein network
  - **IPA103** — IPA canonical pathway results for the 103-protein network
- **string_network_coordinates.tsv** — Node layout coordinates for the STRING protein interaction network displayed in Figure 2, downloaded from the STRINGdb web portal. Used to reproduce the publication-quality network visualization in R
- **FIG3BLOODRNA.xlsx** — Raw and processed data for Figure 3 (whole blood transcriptomics & deconvolution).
- Tabs include:
  - **TTRvsNRBASELINE, TTRvsNRPREPOST** — Differential expression top tables at baseline and pre–post treatment
  - **IPARvNRBL, IPARvNRPREPOST** — IPA pathway results for baseline and pre–post responder vs. non-responder comparisons
  - **RCTMetadataDeconv** — De-identified participant metadata including Xcell-based immune cell deconvolution estimates and UMAP coordinates
  - **CBCpatient** — Baseline complete blood count (CBC) data for available patients
- **FIG4CYTOKINES.xlsx** — Raw and processed data for Figure 4 (plasma cytokine mixed-effects models).
- Tabs include:
  - **PLASMACYTOKINES** — Longitudinal plasma cytokine concentrations and derived ratios used in the primary analysis, with clinical covariates (MADRS, treatment assignment, timepoints, demographics
  - **CYTOKINE_LOD_DATA** — Experimental standards and limit of detection data for all cytokines in the multiplex assay
  - **EXCLUDED_CYTOKINES** — Cytokines captured by the multiplex assay with excessive readings below LOD, which were excluded from primary analyses (see *Methods/Supplemental Methods*)
  - **COMORBIDMEDS** — Medication and comorbidity data used for anti-inflammatory sensitivity analyses
  - **Clinical Variables** — Additional Demographic and clinical ratings variables for patients with RNA (Figure 2) and cytokine (Figure 3) data. Can be used to replicate Table S4, or for additional analyses if desired.
